# *O*-GlcNAcylation Signal Mediates Proteasome Inhibitor Resistance in Cancer Cells by Stabilizing NRF1

**DOI:** 10.1128/MCB.00252-18

**Published:** 2018-08-15

**Authors:** Hiroki Sekine, Keito Okazaki, Koichiro Kato, M. Morshedul Alam, Hiroki Shima, Fumiki Katsuoka, Tadayuki Tsujita, Norio Suzuki, Akira Kobayashi, Kazuhiko Igarashi, Masayuki Yamamoto, Hozumi Motohashi

**Affiliations:** a Department of Gene Expression Regulation, Institute of Development, Aging and Cancer, Tohoku University, Sendai, Japan; b Division of Oxygen Biology, Tohoku University Graduate School of Medicine, Sendai, Japan; c Department of Biochemistry, Tohoku University Graduate School of Medicine, Sendai, Japan; d Department of Medical Biochemistry, Tohoku University Graduate School of Medicine, Sendai, Japan; e Department of Integrative Genomics, Tohoku Medical Megabank Organization, Tohoku University, Sendai, Japan; f Laboratory of Biochemistry, Department of Applied Biochemistry and Food Science, Faculty of Agriculture, Saga University, Saga, Japan; g Laboratory for Genetic Code, Graduate School of Life and Medical Sciences, Doshisha University, Kyotanabe, Kyoto, Japan

**Keywords:** NRF1, O-GlcNAcylation, OGT, proteasome

## Abstract

Cancer cells often heavily depend on the ubiquitin-proteasome system (UPS) for their growth and survival. Irrespective of their strong dependence on the proteasome activity, cancer cells, except for multiple myeloma, are mostly resistant to proteasome inhibitors. A major cause of this resistance is the proteasome bounce-back response mediated by NRF1, a transcription factor that coordinately activates proteasome subunit genes. To identify new targets for efficient suppression of UPS, we explored, using immunoprecipitation and mass spectrometry, the possible existence of nuclear proteins that cooperate with NRF1 and identified *O*-linked *N*-acetylglucosamine transferase (OGT) and host cell factor C1 (HCF-1) as two proteins capable of forming a complex with NRF1. *O*-GlcNAcylation catalyzed by OGT was essential for NRF1 stabilization and consequent upregulation of proteasome subunit genes. Meta-analysis of breast and colorectal cancers revealed positive correlations in the relative protein abundance of OGT and proteasome subunits. OGT inhibition was effective at sensitizing cancer cells to a proteasome inhibitor both in culture cells and a xenograft mouse model. Since active *O*-GlcNAcylation is a feature of cancer metabolism, our study has clarified a novel linkage between cancer metabolism and UPS function and added a new regulatory axis to the regulation of the proteasome activity.

## Abstract (precis)

Cancer cells often heavily depend on the ubiquitin-proteasome system (UPS) for their growth and survival. Irrespective of their strong dependence on the proteasome activity, cancer cells, except for multiple myeloma, are mostly resistant to proteasome inhibitors.

## INTRODUCTION

The ubiquitin-proteasome system (UPS) is involved in various biological processes and plays an important role in the maintenance of cellular proteostasis. The proteasome, which is an ATP-dependent protease that degrades polyubiquitinated substrates, is a 26S multiprotein complex composed of the 20S subcomplex with proteolytic activity and the 19S subcomplex with regulatory activity (1). The overall activity of the proteasome is regulated mainly at two levels: the transcription of proteasome subunit genes and the assembly of proteasome subunit proteins (1, 2). Dysregulation of UPS has been shown to underlie various disorders, including cancers, and modulation of proteasome function has been applied to ameliorate diseases that are associated with altered activity of UPS (3). Cancers often exhibit increased UPS activity and heavily rely on the UPS for proliferation and survival (4–6). However, suppression of UPS by proteasome inhibitors does not produce any satisfactory outcomes in the treatment of cancers except in the case of multiple myeloma (4). A major obstacle is the development of resistance to proteasome inhibitors, resulting from the so-called proteasome bounce-back response, which is a cellular response that restores proteasome activity by transcriptionally activating proteasome subunit genes (2, 4, 7).

Recent studies demonstrated that nuclear factor erythroid 2-related factor 1 (NRF1) is a key transcription factor that is responsible for the induction of proteasome subunit genes by proteasome inhibition and acts as a rheostat of proteasome activity *in vivo* (8–10). NRF1 belongs to the cap'n'collar (CNC) family of transcription factors, possessing a well-conserved basic-region leucine zipper (bZip) motif, and it heterodimerizes with small MAF (sMAF) proteins, which are members of another bZip transcription factor family (11, 12). Neural tissue-specific *Nrf1* knockout mice exhibit abnormal accumulation of polyubiquitinated proteins in the brain, supporting an essential role of NRF1 in the maintenance of proteasome function (13, 14).

NRF1 is initially synthesized as an endoplasmic reticulum (ER) transmembrane protein possessing a long C-terminal portion with N-linked glycosylation in the ER lumen and a short N-terminal portion in the cytoplasm (15, 16). Under normal conditions, NRF1 is subjected to ER-associated degradation (ERAD); the luminal portion of NRF1 is retrotranslocated to the cytoplasm by p97/VCP, followed by its deglycosylation and ubiquitination for degradation (15–21). When cells are exposed to proteasome inhibitors, NRF1 is stabilized and cleaved by DDI-2 protease, resulting in a release of processed NRF1 from the ER into the nucleus and transcriptional activation of proteasome subunit genes (22–24). Thus, ERAD is recognized as a critical node in the regulation of NRF1 activity. In contrast, a post-ER mechanism of NRF1 regulation has been described as a stability control by Fbw7- or β-TrCP-dependent UPS (25, 26).

*O*-linked *N*-acetylglucosamine (*O*-GlcNAc) is a posttranslational modification at serine or threonine residues of target proteins in the nucleus and cytoplasm (27). As serine and threonine residues are also target sites of phosphorylation, *O*-GlcNAcylation is considered to compete with phosphorylation for certain Ser/Thr target sites (28). *O*-linked *N*-acetylglucosamine transferase (OGT) is an enzyme that catalyzes the addition of a GlcNAc moiety by using UDP-GlcNAc as a substrate. UDP-GlcNAc is synthesized through the hexosamine biosynthesis pathway (HBP), and its amount is influenced by nutritional conditions. For example, increased availability of glucose elevates cellular UDP-GlcNAc levels and consequently promotes protein *O*-GlcNAcylation. Recent studies have described enhancement of protein *O*-GlcNAcylation in a wide range of cancers, such as breast and colorectal cancers, and *O*-GlcNAcylation has attracted attention as a new target in cancer treatments (29). However, relevant targets of *O*-GlcNAcylation in cancers have yet to be fully described.

To obtain a clue to overcoming the resistance to proteasome inhibitors acquired by cancer cells, we explored the possible existence of a new putative pathway that controls the magnitude or threshold of the proteasome bounce-back response by investigating NRF1-interacting proteins in the nucleus, hoping to clarify a post-ER regulation of NRF1 activity. We identified OGT/host cell factor C1 (HCF-1) complex, which turned out to be essential for the proteasome inhibitor-induced induction of proteasome subunit genes through increasing the protein level of NRF1. Meta-analysis of The Cancer Genome Atlas (TCGA) data sets of breast and colorectal cancers revealed positive correlations in protein abundance between OGT and proteasome subunits. *OGT* knockdown enhanced the anticancer effect of proteasome inhibitor in both culture cells and a xenograft mouse model. This study has revealed a critical contribution of *O*-GlcNAcylation in cancer cells to maintaining proteasome activity via NRF1 protein stabilization.

## RESULTS

### The OGT/HCF-1 complex interacts with NRF1.

**FIG 1:**
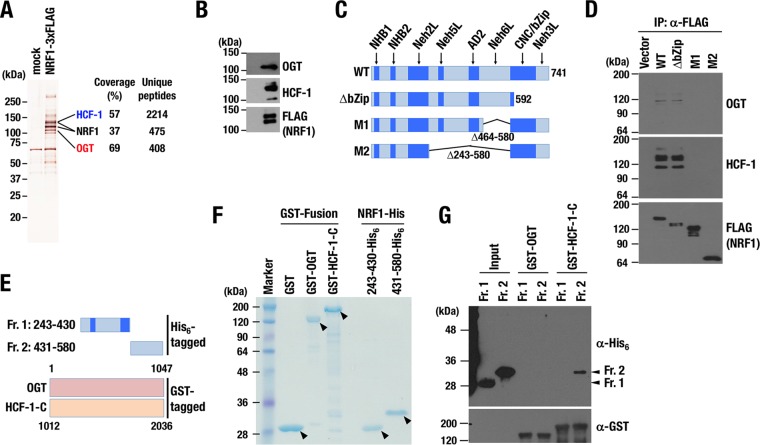
NRF1 interacts with the OGT/HCF-1 complex. (A) Silver staining of NRF1 nuclear complex. Nuclear extracts of 293F cells expressing NRF1-3×FLAG and those with an empty vector (mock) were pulled down with an anti-FLAG antibody. (B) Detection of OGT and HCF-1 proteins in NRF1 nuclear complex shown in panel A by immunoblot analysis. (C) Constructs of 3×FLAG fusion proteins of NRF1 deletion mutants. 3×FLAG-NRF1 WT (1–741), 3×FLAG-NRF1 ΔbZip (1–592), 3×FLAG-NRF1 M1 (Δ464–580), and 3×FLAG-NRF1 M2 (Δ243-580) were expressed in 293F cells and immunoprecipitated. (D) Detection of OGT and HCF-1 proteins interacting with NRF1 and its mutant molecules. Nuclear extracts of 293F cells expressing NRF1 and its mutant molecules were pulled down with an anti-FLAG antibody. Immunoprecipitated samples were subjected to immunoblot analysis with antibodies against OGT, HCF-1, and the FLAG tag. (E) Constructs of His_6_-tagged proteins of NRF1 mutants and GST fusion proteins of OGT and the C-terminal half of HCF-1 (HCF-1-C). Also shown are NRF1-Neh5L/AD2-His_6_ (Fr. 1; 243–430) and NRF1-Neh6L-His_6_ (Fr. 2; 431–580). (F) Coomassie brilliant blue staining of GST fusion proteins and His_6_-tag proteins. GST-OGT, GST–HCF-1-C, NRF1-Neh5L/AD2-His_6_ (Fr. 1; 243–430), and NRF1-Neh6L-His_6_ (Fr. 2; 431–580) were bacterially expressed and purified. Arrowheads indicate purified recombinant fusion proteins. (G) GST pulldown assay of NRF1-His_6_ fragments using GST-OGT and GST–HCF-1-C. NRF1 fragments were detected using an anti-His_6_ antibody, and OGT and HCF-1 were detected using an anti-GST antibody.

To explore the possible existence of a pathway involved in the proteasome inhibitor-mediated bounce-back response, we explored factors participating in a post-ER regulation of NRF1 activity by identifying proteins that interact with NRF1 in the nucleus. 293F cells that stably express 3×FLAG-tagged NRF1 (NRF1-3×FLAG) were established, and NRF1-containing protein complexes were purified from their nuclear extracts with anti-FLAG antibody (Fig. 1A). The purified proteins were subjected to liquid chromatography-tandem MS (LC-MS/MS) analysis. Among them, we identified OGT, an enzyme that catalyzes conjugation of *O*-GlcNAc to target proteins, and HCF-1, which is known as an OGT-binding partner and a chromatin-binding regulator (30–32), as novel binding proteins of NRF1. Immunoblot analysis confirmed the interaction of OGT and HCF-1 with NRF1 (Fig. 1B). Previous studies showed that OGT regulates many biological processes through *O*-GlcNAc modification of target proteins (27, 33) and that active *O*-GlcNAcylation is closely related to cancer malignancy (34–36). Expecting that *O*-GlcNAcylation modulated the proteasome bounce-back response by regulating NRF1 activity, we focused on the contribution of the OGT/HCF-1 complex to NRF1 function.

### NRF1 Neh6L domain directly interacts with the C-terminal region of HCF-1.

To characterize the interaction between NRF1 and the OGT/HCF-1 complex, we determined which domain of NRF1 mediated the interaction with the OGT/HCF-1 complex. Several functional domains and motifs have been defined in NRF1, as shown in Fig. 1C (20). We generated several deletion mutant molecules of NRF1 with FLAG tags (Fig. 1C) and expressed them in 293F cells. Immunoprecipitation assays using the anti-FLAG antibody showed that NRF1 M1 and M2 mutants, both lacking the Neh6L domain, failed to interact with OGT or HCF-1 proteins (Fig. 1D). The NRF1 ΔbZip mutant interacted with both OGT and HCF-1, indicating that CNC/bZip and Neh3L domains are dispensable for the interaction. Thus, we concluded that the OGT/HCF-1 complex interacts with NRF1 via the Neh6L domain.

To examine whether NRF1 directly interacts with the OGT/HCF-1 complex, we generated recombinant NRF1 fragments with His_6_ tags (His_6_-NRF1-Neh5L/AD2 [243–430] and His_6_-NRF1-Neh6L [431–580]) and glutathione *S*-transferase (GST) fusion proteins with full-length OGT (GST-OGT) and the C-terminal half of HCF-1 (GST–HCF-1-C [1012–2036]) (Fig. 1E and F). GST-OGT did not pull down either Neh5L/AD2 (fragment 1 [Fr. 1]) or Neh6L (Fr. 2), whereas GST–HCF-1-C interacted with Neh6L (Fig. 1G), indicating that NRF1 directly interacts with the HCF-1 C-terminal region via Neh6L.

### The OGT/HCF-1 complex is essential for the proteasome inhibitor-mediated bounce-back response.

**FIG 2:**
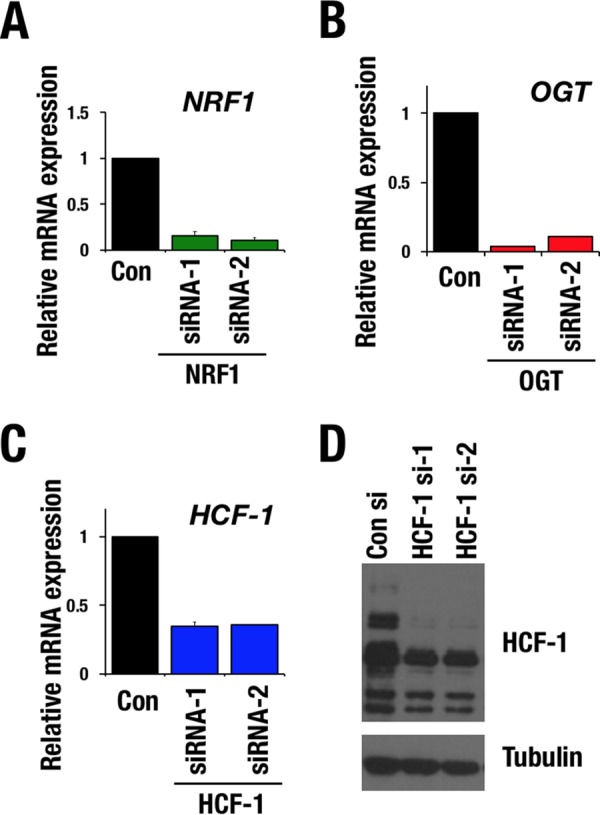
Knockdown efficiency of *NRF1*, *OGT*, and *HCF-1* in HeLa cells. (A to C) Relative mRNA levels of *NRF1* (A), *OGT* (B), and *HCF-1* (C) in HeLa cells that were transfected with control (Con), *NRF1*, *OGT*, or *HCF-1* siRNA. Values were normalized to HPRT values. Normalized values of control cells were set to 1. Averages and SD were calculated from triplicate samples. (D) Immunoblot analysis of HCF-1 in HeLa cells that were transfected with control siRNA or *HCF-1* siRNAs. Tubulin was used as a loading control.

**FIG 3:**
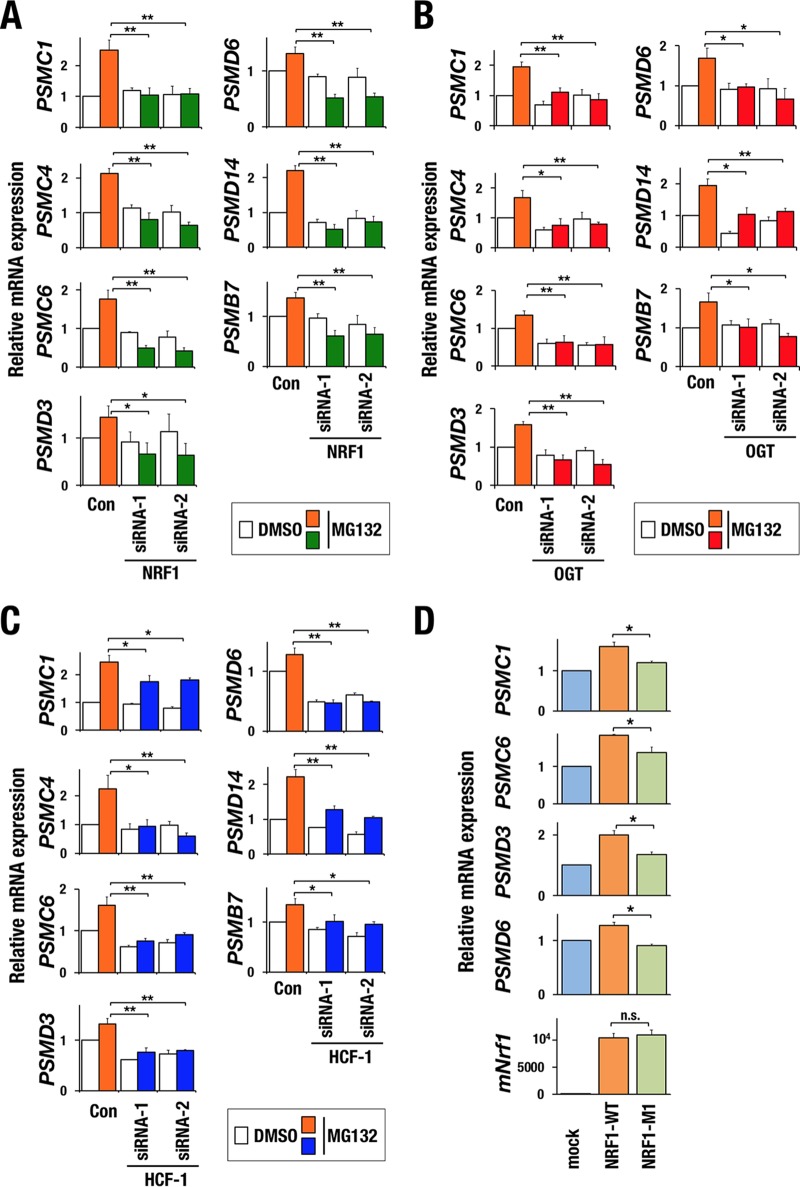
OGT/HCF-1 complex is required for activation of proteasome subunit genes in response to proteasome inhibition. (A to C) Relative mRNA levels of proteasome subunit genes. HeLa cells were transfected with control siRNA, *NRF1* siRNAs (A), *OGT* siRNAs (B), or *HCF-1* siRNAs (C). After 72 h, the cells were treated with DMSO or 1 μM MG132 for 10 h. Values were normalized to HPRT values. Normalized values of control cells that were treated with DMSO were set to 1. Averages and SD were calculated from triplicate samples. *, *P* < 0.05; **, *P* < 0.01. (D) Relative mRNA levels of proteasome subunit genes. 293F cells were stably transduced with empty vector, 3×FLAG-NRF1-WT, or 3×FLAG-NRF1-M1 expression vector and treated with high-glucose medium for 24 h before harvest. Values were normalized to HPRT values. The normalized values of mock-transduced cells were set to 1. Averages and SD were calculated from triplicate samples. *, *P* < 0.01. n.s., not significant.

To examine whether the OGT/HCF-1 complex is involved in the proteasome bounce-back response by regulating NRF1 activity, we first verified a contribution of NRF1 to the expression of proteasome subunit genes in our experimental setting. Treatment with the proteasome inhibitor MG132 increased the expression levels of proteasome subunit genes in HeLa cells with control short interfering RNA (siRNA), whereas the MG132-induced upregulation was completely abrogated by *NRF1* knockdown (Fig. 2A and 3A). We then examined the contributions of OGT and HCF-1 to the bounce-back response by knocking down each factor (Fig. 2B to D). Knocking down *OGT* attenuated the upregulation of the proteasome subunit genes in response to MG132 (Fig. 3B). Similar results were obtained in *HCF-1* knockdown cells (Fig. 3C). These results indicate that the OGT/HCF-1 complex is required for the proteasome bounce-back response and suggest that the OGT/HCF-1 complex supports the NRF1 activity.

We next examined whether recruitment of the OGT/HCF-1 complex to NRF1 was important for NRF1-mediated transcriptional activation of proteasome subunit genes by utilizing the NRF1 M1 mutant that was incapable of interacting with the OGT/HCF-1 complex (Fig. 1C and D). Proteasome subunit genes were upregulated by exogenous wild-type NRF1; however, the upregulation was not obvious in the case of the NRF1 M1 mutant (Fig. 3D), indicating that interaction of NRF1 with the OGT/HCF-1 complex is necessary for NRF1-mediated transcriptional activation.

### HCF-1 is required for chromatin binding to NRF1 at promoter regions of proteasome subunit genes.

NRF1 has been shown to activate proteasome subunit genes by binding to their promoter regions (8, 9, 37). To comprehensively assess the role of NRF1 in transcriptional regulation, we conducted chromatin immunoprecipitation sequencing (ChIP-seq) analysis in HeLa cells that were treated with MG132 by using NRF1 antibody. Consistent with previous reports, NRF1 was localized at promoter regions of almost all proteasome subunit genes (see Fig. S1A and B in the supplemental material).

**FIG 4:**
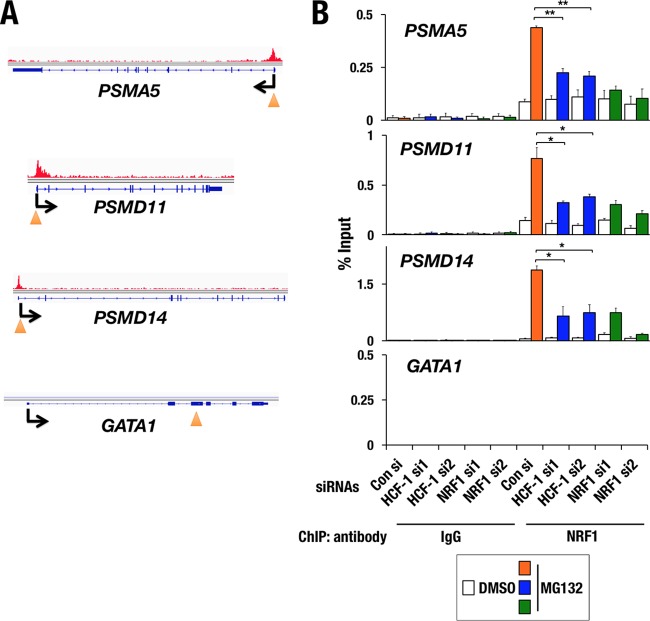
OGT/HCF-1 complex is required for chromatin binding of NRF1. (A) NRF1 ChIP-seq binding sites in *PSMA5*, *PSMD11*, *PSMD14*, and *GATA-1* gene loci. An arrowhead indicates the position of a primer set that was used for the ChIP assay within each target gene locus. (B) Quantitative ChIP assay at each gene locus in HeLa cells that were treated with control siRNA, two distinct siRNAs specific for *HCF-1* (*HCF-1*-si1 and *HCF-1*-si2), or two distinct siRNAs specific for *NRF1* (*NRF1*-si1 and *NRF1*-si2). Chromatin localization of NRF1 was examined in each sample that was treated with 1 μM MG132 or DMSO for 4 h. The values show the enrichment of immunoprecipitated DNA relative to input DNA. Averages and SDs were calculated from triplicate samples. *, *P* < 0.01; **, *P* < 0.001.

To understand how the OGT/HCF-1 complex regulates the intranuclear function of NRF1, we knocked down the expression of HCF-1, which directly interacts with NRF1 and is known to be a chromatin-binding regulator (32), and examined NRF1 binding to the promoters of the proteasome subunit genes. In HeLa cells transfected with control siRNA, as observed in the ChIP-seq analysis, MG132 treatment induced robust binding of NRF1 to the promoter regions of the representative proteasome subunit genes *PSMA5*, *PSMD11*, and *PSMD14* but not to a negative-control locus, *GATA1* (Fig. 4). In contrast, RNA interference of HCF-1 reduced the MG132-induced chromatin binding of NRF1 (Fig. 4), suggesting that the OGT/HCF-1 complex is essential for chromatin binding of NRF1 at proteasome subunit gene loci.

### The OGT/HCF-1 complex is required for NRF1 protein accumulation in response to proteasome inhibition.

**FIG 5:**
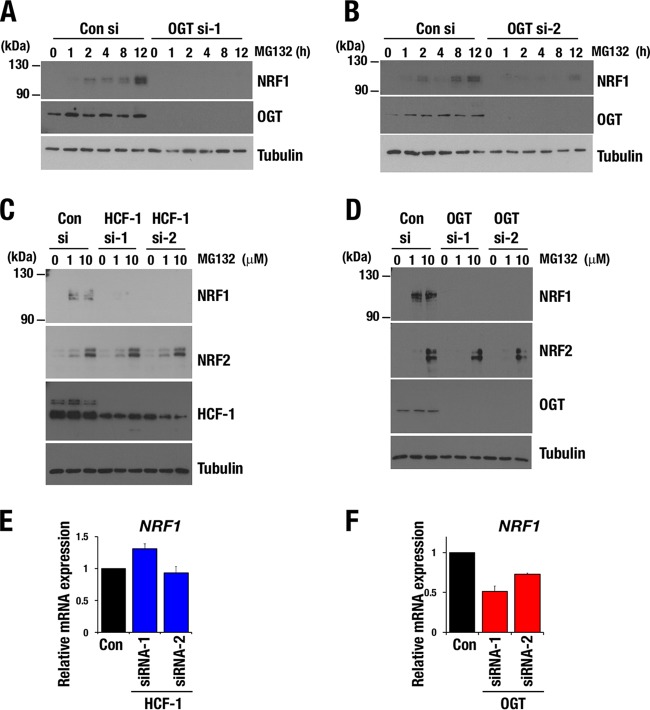
OGT/HCF-1 complex is required for accumulation of NRF1 protein. (A and B) NRF1 accumulation in response to MG132 treatment in HeLa cells. HeLa cells that were transfected with control siRNA, *OGT* siRNA-1 (A), or *OGT* siRNA-2 (B) were treated with 1 μM MG132 at 72 h after transfection. Whole-cell extracts were prepared at the indicated time points after treatment with MG132. Reduction of OGT protein was verified in *OGT* siRNA-treated cells, and tubulin was used as a loading control. (C and D) NRF1 and NRF2 accumulation in response to a proteasome inhibitor. MDA-MB-231 cells that were transfected with control siRNA, *HCF-1* siRNAs (C), or *OGT* siRNAs (D) were treated with 1 or 10 μM MG132 at 72 h after the transfection and cultured for another 4 h before harvest. Whole-cell extracts were prepared and subjected to immunoblot analysis with antibodies against NRF1, NRF2, HCF-1 (C), OGT (D), and tubulin. (E and F) Relative mRNA levels of *NRF1* in MDA-MB-231 cells that were transfected with control siRNA, *HCF-1* siRNAs (E), or *OGT* siRNAs (F). Values were normalized to HPRT values. Normalized values of control cells were set to 1. Averages and SD were calculated from the results of triplicate samples.

To understand how the OGT/HCF-1 complex contributes to the chromatin binding of NRF1, we examined the accumulation of endogenous NRF1 protein in response to proteasome inhibition after knocking down *OGT* or *HCF-1*. We found that *OGT* knockdown in HeLa cells abrogated NRF1 protein accumulation that was induced by MG132 (Fig. 5A and B). *HCF-1* or *OGT* knockdown in a breast cancer cell line, MDA-MB231 cells, also abolished the MG132-induced elevation of NRF1 protein levels (Fig. 5C and D), which were not necessarily correlated with NRF1 mRNA levels. Abundance of NRF1 mRNA was not altered by *HCF-1* knockdown but was reduced by *OGT* knockdown (Fig. 5E and F). The results indicate that HCF-1, which recruits OGT to NRF1, is involved in the posttranscriptional regulation of NRF1 and mediates NRF1 protein accumulation in response to proteasome inhibition. OGT might be involved in the transcriptional regulation of *NRF1* in an HCF-1-independent manner.

**FIG 6:**
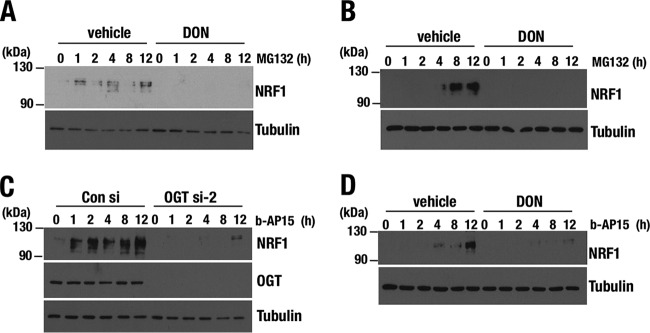
Hexosamine biosynthesis pathway is required for accumulation of NRF1 protein. (A and B) NRF1 accumulation in response to MG132 treatment in HeLa (A) and MDA-MB231 (B) cells. HeLa and MDA-MB231 cells were pretreated with 100 μM DON for 24 h and treated with 1 μM MG132. Whole-cell extracts were prepared at the indicated time points after the cells were treated with MG132. Tubulin was used as a loading control. (C and D) NRF1 accumulation in response to b-AP15 treatment in MDA-MB231 cells. At 72 h after transfection with *OGT* siRNA (C) or 24 h after 100 μM DON treatment (D), whole-cell extracts were prepared at the indicated time points after treatment with b-AP15. Tubulin was used as a loading control.

To examine the significance of the enzymatic activity of OGT, we inhibited *O*-GlcNAcylation by suppressing the hexosamine biosynthesis pathway (HBP) and limiting the availability of UDP-GlcNAc, a substrate of *O*-GlcNAcylation. An HBP-suppressing reagent, 6-diazo-5-oxo-l-norleucine (DON), abrogated MG132-induced NRF1 accumulation in HeLa cells as well as in MDA-MB-231 cells (Fig. 6A and B). Thus, *O*-GlcNAcylation catalyzed by OGT is required for the NRF1 accumulation that is induced by proteasome inhibition.

A previous study demonstrated that OGT inhibits proteasome activity by *O*-GlcNAcylating a subunit of the 19S proteasome subcomplex (38), implying that proteasome activity, especially that of the 19S proteasome, was increased in the *OGT* knockdown cells. To evaluate the possibility that *OGT* knockdown enhanced 19S proteasome activity and subsequently inhibited NRF1 accumulation, we used the proteasome inhibitor b-AP15, which specifically targets the 19S proteasome subunit (39). *OGT* knockdown and DON treatment both abolished b-AP15-induced accumulation of NRF1 in a manner similar to that induced by MG132, which targets the 20S subunit (Fig. 6C and D). These results suggest that decreased *O*-GlcNAcylation limits NRF1 accumulation independently of influencing proteasomal activity. To further exclude the possibility that a decrease in the amount of the OGT/HCF-1 complex potentiated proteasome activity and nonspecifically inhibited NRF1 accumulation, we investigated the protein level of NRF2, which is predominantly regulated by the UPS (40), in MDA-MB231 cells by *HCF-1* or *OGT* knockdown. Knocking down HCF-1 or OGT did not affect NRF2 protein accumulation in response to MG132 (Fig. 5C and D). These results suggest that *O*-GlcNAcylation mediated by the OGT/HCF-1 complex is specifically critical to NRF1 accumulation in response to proteasome inhibition. We thus concluded that the OGT/HCF-1 complex supports the activation of proteasome subunit genes by enhancing the accumulation and subsequent chromatin binding of NRF1 when proteasome activity is compromised.

### Enhanced cellular *O*-GlcNAcylation promotes NRF1 protein accumulation.

**FIG 7:**
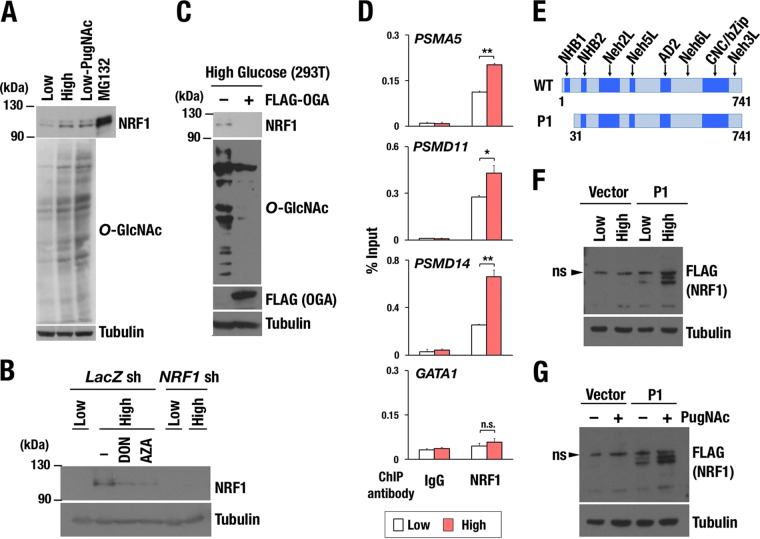
Increased activity of cellular *O*-GlcNAcylation enhances accumulation of NRF1 protein. (A) Effect of *O*-GlcNAcylation on the protein level of endogenous NRF1. Whole-cell extracts were prepared from Hep3B cells that were cultured in medium containing 1.5 g/ml glucose (low), 4.5 g/ml glucose (high), or 1.5 g/ml glucose with 100 μM PugNAc (Low-PugNAc) for 24 h and subjected to immunoblot analysis with antibodies against NRF1, *O*-GlcNAc peptides, and tubulin. Hep3B cells that were treated with 10 μM MG132 were used as a positive control in NRF1 detection. (B) Effect of HBP inhibitors on the protein levels of endogenous NRF1. Whole-cell extracts were prepared from 293T cells expressing *lacZ* shRNA or *NRF1* shRNA. Cells were cultured in low- or high-glucose medium with or without HBP inhibitors, 100 μM DON, or 100 μM AZA for 24 h before harvest. The protein extracts were subjected to immunoblot analysis with antibodies against NRF1 and tubulin. (C) Effect of OGA expression on the protein levels of endogenous NRF1. 293T cells were transfected with an empty or a FLAG-OGA expression vector. At 24 h after transfection, the cells were cultured in high-glucose medium for another 24 h and then harvested. Whole-cell extracts were prepared and subjected to immunoblot analysis with antibodies against NRF1, *O*-GlcNAc peptides, the FLAG tag, and tubulin. (D) Quantitative ChIP assay at the *PSMA5*, *PSMD11*, *PSMD14*, and *GATA-1* gene loci in HeLa cells. Chromatin localization of NRF1 was examined in each sample that was treated with low or high glucose for 24 h. The values show the enrichment of immunoprecipitated DNA relative to input DNA. Averages and SD were calculated from triplicate samples. *, *P* < 0.05; **, *P* < 0.01. ns, not significant. (E) Constructs of 3×FLAG fusion proteins of NRF1 WT and P1 mutant (Δ30). (F and G) Accumulation of nuclear NRF1 (NRF1 P1) by enhancing cellular *O*-GlcNAcylation. 293F cells expressing 3×FLAG-NRF1 P1 were cultured in the medium containing low or high glucose (F) and treated with 100 μM PugNAc or left untreated (G). After 24 h, whole-cell extracts were prepared and subjected to immunoblot analysis with antibodies against the FLAG tag and tubulin.

We next investigated whether enhancement of cellular *O*-GlcNAcylation increased the endogenous abundance of NRF1 protein, because metabolic reprogramming of cancer cells often supports high levels of *O*-GlcNAcylation (33–36, 41). Cellular *O*-GlcNAcylation was facilitated by increasing the availability of extracellular glucose, from which UDP-GlcNAc is generated through HBP, or by inhibiting OGA, which catalyzes the removal of GlcNAc from proteins. We treated Hep3B cells with either high-glucose medium or the OGA inhibitor PugNAc to induce cellular protein *O*-GlcNAcylation. Both treatments robustly increased NRF1 protein accumulation (Fig. 7A). Suppression of HBP by the specific inhibitors DON and azaserine (AZA) attenuated the effect of high glucose on NRF1 accumulation (Fig. 7B). Overexpression of OGA inhibited the high-glucose-dependent promotion of cellular *O*-GlcNAcylation and NRF1 protein accumulation (Fig. 7C). These results indicate that enhanced *O*-GlcNAcylation increases endogenous protein levels of NRF1.

To verify whether elevated *O*-GlcNAcylation increased NRF1 binding to promoters of proteasome subunit genes, we performed the ChIP assay. In HeLa cells, high-glucose treatment facilitated chromatin binding of NRF1 to the promoter regions of the representative proteasome subunit genes *PSMA5*, *PSMD11*, and *PSMD14* but not to the negative-control locus *GATA1* (Fig. 7D), suggesting that NRF1 contributes to the activation of the proteasome subunit genes when it is accumulated in response to enhanced cellular *O*-GlcNAcylation.

To clarify whether the processed form of NRF1 responded to the facilitation of *O*-GlcNAcylation, we used 293F cells that expressed the NRF1 P1 mutant, which lacks an ER retention sequence and mainly exists in the nucleus (Fig. 7E). The level of NRF1 P1 protein was robustly increased in response to high-glucose conditions (Fig. 7F) as well as PugNAc treatment (Fig. 7G). Thus, *O*-GlcNAcylation is able to act on processed NRF1 to promote its accumulation. Based on this result and intracellular localization of OGT/HCF-1 in cytoplasm and nucleus but not in ER, we surmise that NRF1 is *O*-GlcNAcylated in the nucleus or cytoplasm after being processed from ER. Namely, *O*-GlcNAcylation contributes to post-ER regulatory mechanisms of NRF1 activity.

### NRF1 is *O*-GlcNAcylated at serine residues that are critical for phosphorylation and interaction with β-TrCP.

**FIG 8:**
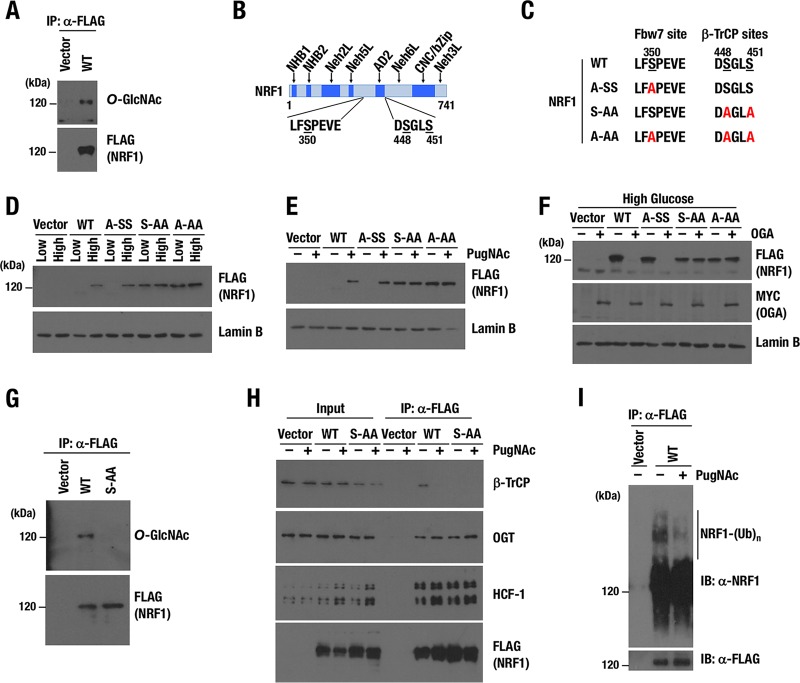
Two serine residues, S448/S451, are critical for *O*-GlcNAcylation of NRF1. (A) Detection of *O*-GlcNAcylation of NRF1 protein. Nuclear extracts of 293T cells expressing NRF1-3×FLAG were prepared and then pulled down with an anti-FLAG antibody. Immunoprecipitated samples were subjected to immunoblot analysis with antibodies against *O*-GlcNAc peptides and the FLAG tag. (B) Motifs in NRF1, which mediate interaction with Fbw7 (left) and β-TrCP (right). Serine residues that were replaced with alanine in subsequent experiments are underlined. The numbers beneath the underlines denote positions of the serine residues in the amino acid sequence of the NRF1 protein. (C) Summary of serine-to-alanine substitutions in the two motifs of NRF1. (D and E) Nuclear accumulation of NRF1 and its mutant molecules in response to enhanced cellular *O*-GlcNAcylation induced by high-glucose condition (D) or PugNAc treatment (E). 293T cells expressing wild-type or mutant NRF1-3×FLAG (WT, A-SS, S-AA, and A-AA) were cultured in low- or high-glucose culture medium (D) or with or without 100 μM PugNAc (E) for 24 h. Nuclear extracts were prepared and subjected to immunoblot analysis with antibodies against the FLAG tag and lamin B. (F) Effects of OGA expression on the protein levels of NRF1 and its mutant molecules. 293T cells expressing wild-type or mutant NRF1-3×FLAG were transfected with an empty or a Myc-OGA expression vector. At 24 h after transfection, the cells were cultured in high-glucose medium for another 24 h and harvested. Nuclear extracts were prepared and subjected to immunoblot analysis with antibodies against the FLAG tag, Myc tag, and lamin B. (G) Identification of critical serine residues for *O*-GlcNAcylation of the NRF1 protein. Nuclear extracts of 293T cells expressing the WT or S-AA mutant of NRF1-3×FLAG were prepared and pulled down with an anti-FLAG antibody. Immunoprecipitated samples were subjected to immunoblot analysis with antibodies against *O*-GlcNAc peptides and the FLAG tag. (H) Effects of PugNAc on protein interactions with NRF1. 293T cells that were stably transfected with an empty vector or an expression vector of the WT or S-AA mutant of NRF1-3×FLAG were pretreated with 100 μM PugNAc or left untreated for 24 h before nuclear extracts were prepared for immunoprecipitation with an anti-FLAG antibody. Immunoprecipitated proteins were subjected to immunoblot analysis with antibodies against β-TrCP, OGT, HCF-1, and the FLAG tag. (I) Effects of PugNAc on ubiquitination of NRF1. 293F cells that were stably transfected with an empty vector or an expression vector of NRF1-3×FLAG were pretreated with or without 100 μM PugNAc for 24 h and incubated with 10 μM MG132 for another 4 h before whole-cell extracts were prepared for immunoprecipitation with an anti-FLAG antibody. Immunoprecipitated proteins were subjected to immunoblot analysis with antibodies against NRF1 and the FLAG tag.

Recent reports have described that many transcription factors are *O*-GlcNAcylated and functionally modified (42). Because transcriptional activation of proteasome subunit genes by NRF1 requires the OGT/HCF-1 complex to be recruited to the CNC-bZip transcription factor via its Neh6L domain (Fig. 3D), we hypothesized that NRF1 is *O*-GlcNAcylated by OGT and that this modification enables NRF1 to activate the target genes. To detect *O*-GlcNAcylation of NRF1, we introduced NRF1-3×FLAG in 293T cells, and a soluble nuclear fraction was prepared. FLAG-tagged NRF1 was *O*-GlcNAcylated sufficiently by endogenous OGT (Fig. 8A).

We moved on to identify amino acids in NRF1 that are the targets for *O*-GlcNAcylation. Previous reports stated that NRF1 interacts with Fbw7 and β-TrCP ubiquitin E3 ligases, resulting in the ubiquitination and degradation of NRF1 (25, 26). Fbw7 and β-TrCP have been shown to recognize phosphorylated serine residues of NRF1, S350 and S448/S451, respectively (Fig. 8B and C) (25, 26). As *O*-GlcNAcylation is considered to compete with phosphorylation for Ser/Thr target sites (27), we hypothesized that *O*-GlcNAcylation of these serine residues stabilizes NRF1 by antagonizing their phosphorylation and consequently protecting NRF1 from the Fbw7- and β-TrCP-mediated degradation. To prove this hypothesis, S350 and S448/S451 were replaced with alanine, and three NRF1 mutant molecules, A-SS, S-AA, and A-AA, were constructed (Fig. 8B and C). FLAG-tagged NRF1 (WT) and its mutant molecules (A-SS, S-AA and A-AA) were stably expressed in 293T cells.

To examine the importance of the serine residues in *O*-GlcNAcylation-induced stabilization of NRF1, we treated these 293T cells with high glucose or PugNAc, both of which enhance *O*-GlcNAcylation. The NRF1 WT and A-SS proteins were detected in nuclear extracts of the cells treated with high glucose but not in those treated with low glucose (Fig. 8D). In contrast, S-AA and A-AA mutants were accumulated even under the low-glucose condition, and the high-glucose treatment did not increase these protein levels any further (Fig. 8D). Similarly, the NRF1 WT and A-SS protein levels were increased in response to PugNAc treatment, but the S-AA and A-AA mutant proteins did not show any further increase (Fig. 8E). These results suggest that S448/S451 are required for the destabilization of NRF1 under low *O*-GlcNAcylation activity and the stabilization of NRF1 in response to the enhancement of cellular *O*-GlcNAcylation.

Conversely, decreased cellular *O*-GlcNAcylation by overexpression of OGA inhibited the high-glucose-induced accumulation of the NRF1 WT and A-SS proteins (Fig. 8F). Alanine substitution of S448/S451 abrogated the response to OGA, suggesting again that S448/S451 are required for NRF1 destabilization under low *O*-GlcNAcylation activity. Indeed, *O*-GlcNAcylation was dramatically suppressed in the NRF1 S-AA mutant compared with that of wild-type NRF1 (Fig. 8G), indicating that S448/S451 are essential for NRF1 *O*-GlcNAcylation and suggesting that S448/S451 are *O*-GlcNAcylation target sites of NRF1.

### *O*-GlcNAcylation of NRF1 interferes with the interaction between NRF1 and β-TrCP ubiquitin E3-ligase.

Because β-TrCP interacts with NRF1 by recognizing phosphorylated S448/S451, it was hypothesized that *O*-GlcNAcylation of S448/S451 disrupts the interaction between NRF1 and β-TrCP. To test this hypothesis, we conducted an immunoprecipitation assay using nuclear extracts of 293T cells expressing the NRF1 WT or A-SS proteins. The cells were treated with 1 μM MG132 for 4 h in the presence or absence of PugNAc before harvesting. When the cells expressing wild-type NRF1 were used, β-TrCP was detected as an NRF1-interacting protein under the control condition together with OGT and HCF-1, whereas PugNAc treatment remarkably decreased the amount of β-TrCP in the NRF1 complex (Fig. 8H). Thus, we infer that *O*-GlcNAcylation at S448/S451 induced by PugNAc disrupts the association between β-TrCP and NRF1. When cells expressing NRF1 S-AA were used, β-TrCP was not detected in the NRF1 S-AA complex, irrespective of the presence or absence of PugNAc. The alanine substitution can be interpreted as mimicking the effects of *O*-GlcNAcylation by preventing phosphorylation of S448/S451. In agreement with the decreased association between β-TrCP and NRF1 in the presence of PugNAc, polyubiquitination of NRF1 was suppressed in PugNAc-treated cells (Fig. 8I).

The data obtained so far indicated that OGT associates with NRF1 through HCF-1 and catalyzes *O*-GlcNAcylation of NRF1, resulting in the release of β-TrCP from NRF1 and the consequent stabilization of NRF1.

### Protein abundances of OGT and proteasome subunits are positively correlated in breast invasive carcinoma and colorectal adenocarcinoma.

Elevated expression of OGT in cancers has been shown to be advantageous for their tumorigenesis and metastasis (34, 43). Another line of evidence has indicated that certain types of cancers depend heavily on proteasome activity for their aggressive growth (5, 6). These studies suggest that enhanced activities of OGT and proteasome both contribute to cancer malignancy. Because our finding indicates that the increased activity of OGT elevates expression levels of proteasome subunit genes through NRF1 stabilization, we examined whether a correlation exists between the abundance of OGT protein and that of proteasome subunits using the cBioPortal to examine clinical cancer cases that were enrolled in the TCGA database (44, 45).

**FIG 9:**
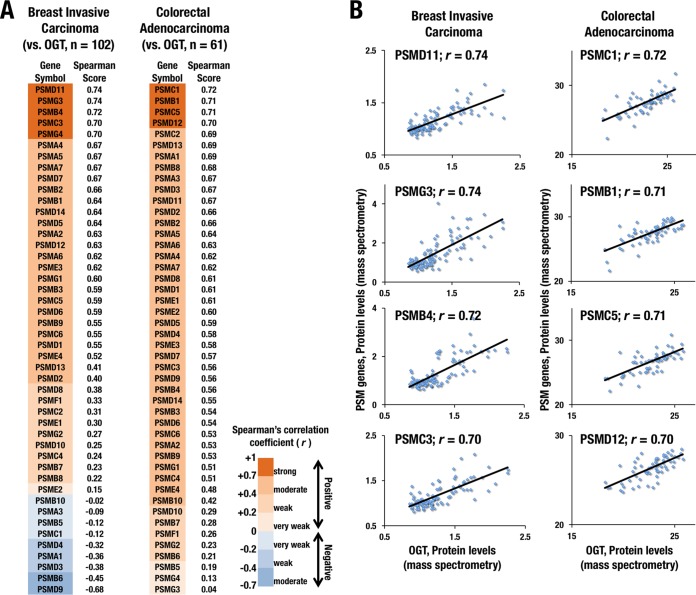
Protein abundances of OGT and proteasome subunits are positively correlated in clinical specimens of breast and colorectal cancers. (A) Correlations of protein abundance of OGT and proteasome subunits in breast invasive carcinoma (left) and colorectal adenocarcinoma (right) are expressed in terms of Spearman's correlation coefficients, which are aligned in declining order. LC-MS/MS data from the TCGA database (http://www.cbioportal.org) were analyzed in the framework of the cBioPortal. (B) Dot plots showing correlations of between-protein abundances of OGT and representative proteasome subunits in breast invasive carcinoma (left) and colorectal adenocarcinoma (right). The strength of the correlations was evaluated with Spearman's rank correlation test.

We analyzed proteomic data sets that were constructed from 102 breast invasive carcinoma cases and found positive correlations between OGT and the majority of proteasome subunits (Fig. 9A and B, left, and Table S1). In 62 cases of colorectal adenocarcinoma that expressed a detectable amount of OGT protein, positive correlation was obtained between OGT and almost all proteasome subunits that were examined (Fig. 9A and B, right, and Table S2). These results strongly suggest that OGT controls proteasome subunit expression in cancer cells.

### Suppression of the OGT/HCF-1 complex improves the efficacy of a proteasome inhibitor as an anticancer drug.

Proteasome inhibitors, including bortezomib, have been used as effective anticancer drugs for certain types of cancers that possess high proteasome activity (4). Previous reports that NRF1 confers resistance to proteasome inhibitors by mediating the proteasome bounce-back response (7–10), in combination with the results that we have obtained so far, strongly suggest that OGT inhibition augments the anticancer effects of proteasome inhibitors.

**FIG 10:**
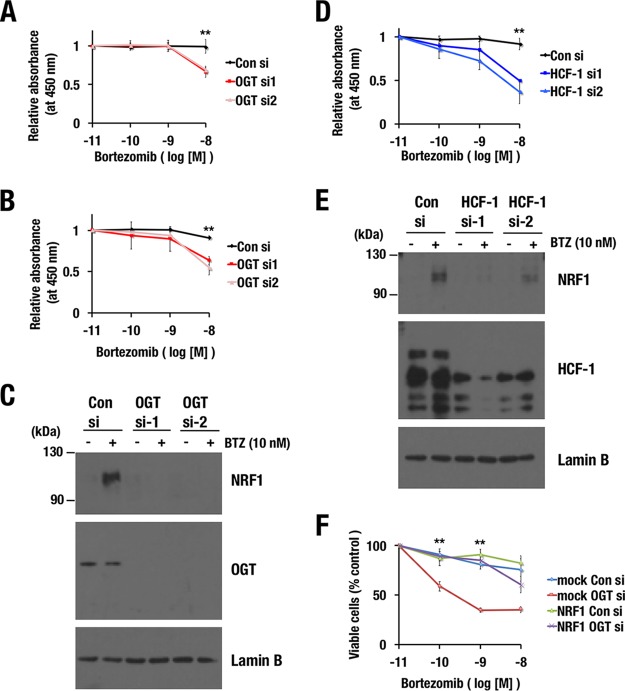
Inhibition of the OGT/HCF-1 complex sensitizes cancer cell lines to a proteasome inhibitor. (A, B, and D) Effects of *OGT* (A and B) or *HCF-1* (D) knockdown on the viability of MDA-MB-231 (A and D) and NCI-H460 (B) cells in the presence of bortezomib. At 24 h after the transfection of control siRNA or *OGT* or *HCF-1* siRNAs, the cells were reseeded in 96-well plates. The next day, the cells were treated with the indicated concentrations of bortezomib for 24 h. Cell viability was monitored using Cell Counting Kit-8. Averages and SD were calculated from quadruplicate samples. Relative absorbances of samples that were treated with 10^−11^ M bortezomib were set to 1. **, *P* < 0.01. (C and E) Endogenous NRF1 accumulation in response to bortezomib treatment in MDA-MB-231 cells. MDA-MB-231 cells that were transfected with control siRNA, *OGT* siRNAs (C), or *HCF-1* siRNAs (E) were treated with 10 nM bortezomib at 72 h after the transfection. After 4 h, nuclear extracts were prepared. Lamin B was used as a loading control. (F) Effects of NRF1 overexpression on *OGT* knockdown-induced sensitization to bortezomib. 293F cells expressing NRF1-3×FLAG or containing an empty vector (mock) were transfected with control siRNA or *OGT* siRNAs. At 24 h after the transfection, the cells were reseeded in 96-well plates. The next day, the cells were treated with the indicated concentrations of bortezomib for 48 h. Cell viability was assessed using a trypan blue exclusion test. Averages and SDs were calculated from the results of three independent experiments. Viable cell numbers for samples that were treated with 10^−11^ M bortezomib were set to 100%. **, *P* < 0.01.

To prove this hypothesis, we knocked down OGT in two different cancer cell lines, MDA-MB-231 and NCI-H460, and examined the amount of bortezomib-induced cell death. Increasing concentrations of bortezomib were applied to control and *OGT* knockdown cells. As we expected, *OGT* knockdown cells were more sensitive to bortezomib than control cells (Fig. 10A to C). *HCF-1* knockdown similarly sensitized MDA-MB-231 cells to bortezomib (Fig. 10D and E). To verify that this sensitization effect was due to the insufficient stabilization of NRF1, NRF1 was exogenously overexpressed in *OGT* knockdown cells. The NRF1-overexpressing cells turned out to be resistant to bortezomib irrespective of the *OGT* knockdown (Fig. 10F), indicating that excessive NRF1 canceled the *OGT* knockdown-mediated sensitization to bortezomib.

**FIG 11:**
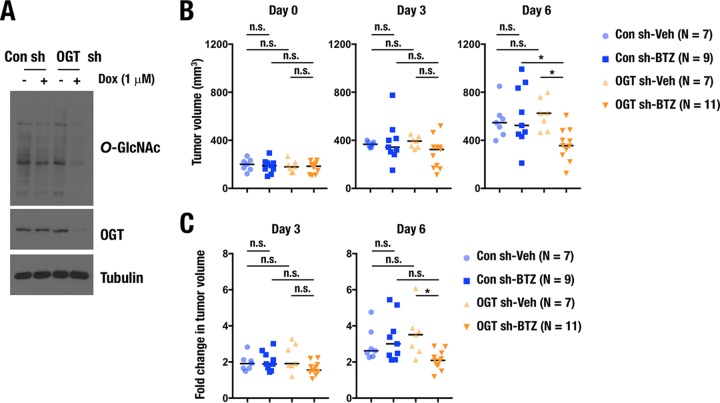
OGT inhibition enhances antitumorigenic activity of a proteasome inhibitor. (A) Immunoblot analysis of O-GlcNAced proteins and OGT in H460 cells expressing doxycycline (Dox)-inducible control shRNA or *OGT* shRNAs. Whole-cell extracts were prepared at 24 h after treatment with 1 μg/ml doxycycline. Tubulin was used as a loading control. (B and C) Effects of bortezomib (BTZ) on the tumor growth of H460 cells expressing control or *OGT* shRNA in xenograft mice model. A total of 1 × 10^6^ H460 cells were subcutaneously transplanted into nude mice. Fifteen days after the transplantation, bortezomib was directly administered into each tumor twice. (B) Tumor volume was calculated on 3 and 6 days after the initial treatment of bortezomib. (C) Fold changes of tumor volumes on day 3 and day 6 were calculated against that on day 0. *, *P* < 0.05. n.s., not significant.

Finally, we examined whether OGT suppression improved efficacy of proteasome inhibitors in a xenograft mouse model. To this end, we established NCI-H460 cells expressing control or *OGT* short hairpin RNAs (shRNAs) in a doxycycline-inducible manner (Fig. 11A). The NCI-H460 cells were subcutaneously transplanted to nude mice, and the shRNAs were induced by treating the mice with doxycycline. After 15 days of transplantation, bortezomib treatment was initiated for tumors whose sizes ranged from 100 to 300 mm^3^. The tumor size of *OGT* knockdown cells was significantly reduced by bortezomib treatment on day 6, whereas that of control cells was not affected (Fig. 11B). When fold changes in the tumor volume on days 3 and 6 against the one on day 0 were calculated, significant suppressive effects of bortezomib were also apparent for the *OGT* knockdown cells but not for the control cells (Fig. 11C). Thus, OGT suppression enhanced the antitumorigenic effect of bortezomib.

From these results, we concluded that *O*-GlcNAcylation is required for the NRF1-dependent proteasome bounce-back response and that OGT inhibition sensitizes cancer cells to proteasome inhibitors by antagonizing NRF1 stabilization.

## DISCUSSION

**FIG 12:**
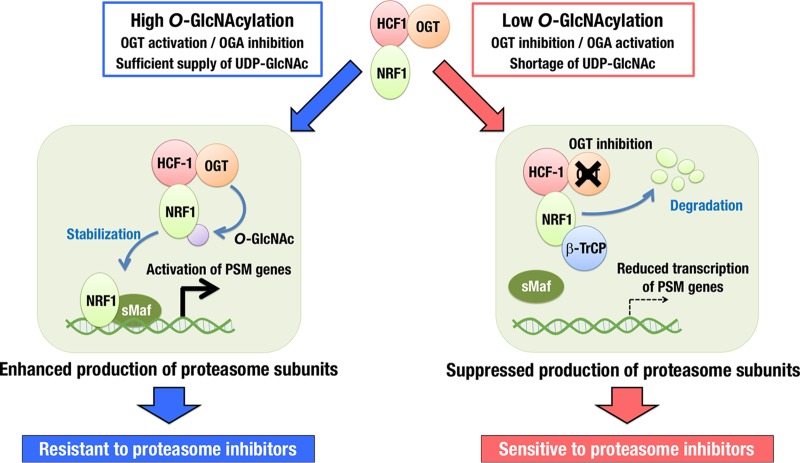
Schematic illustration of *O*-GlcNAcylation switching on and off the activity of NRF1 for transcription of proteasome subunit (PSM) genes. With low *O*-GlcNAcylation activity, NRF1 preferentially interacts with β-TrCP and becomes ubiquitinated and degraded. With high *O*-GlcNAcylation activity, which is often observed in cancer cells, NRF1 is *O*-GlcNAcylated by its binding partner OGT/HCF-1 complex. *O*-GlcNAcylation of NRF1 disrupts the association between β-TrCP and NRF1, resulting in the enhancement of NRF1 accumulation, elevation of the PSM gene expression, and resistance to proteasome inhibitors. OGT inhibition effectively sensitizes cancer cells to proteasome inhibitors by suppressing the NRF1-mediated expression of PSM genes.

Our study has revealed that an *O*-GlcNAcylation signal makes a critical contribution to the proteasome bounce-back response via increasing NRF1 stability (Fig. 12). Protein *O*-GlcNAcylation status is determined by the balance between OGT and OGA activities as well as the availability of the substrate UDP-GlcNAc. When OGT activity predominates with a sufficient supply of UDP-GlcNAc, *O*-GlcNAcylated NRF1 releases β-TrCP ubiquitin E3-ligase from the NRF1 complex, resulting in the stabilization of NRF1 and upregulation of proteasome subunit genes. Thus, the *O*-GlcNAcylation level is critical for the maintenance of proteasome activity by regulating NRF1 stability. Our discovery of the interaction between NRF1 and the OGT/HCF-1 complex has added a new regulatory axis to the proteasome bounce-back response and identified OGT as a new therapeutic target for sensitizing cancer cells to proteasome inhibitors.

From the viewpoint of cancer metabolism, facilitation of glycolysis and glutaminolysis in cancer cells provides a high availability of UDP-GlcNAc, resulting in an advantageous metabolic environment for *O*-GlcNAcylation (41). Thus, in cancer cells, OGT is likely to exert its full activity without being restricted by substrate limitation, which would positively correlate the OGT protein level with the *O*-GlcNAcylation activity, the NRF1 protein level, and the expression levels of proteasome subunit genes. This scenario may explain the strong positive correlations between protein abundances of OGT and most of the proteasome subunits in breast and colorectal cancers (Fig. 9). We propose that metabolic reprogramming of cancers allows highly expressed OGT to maximally contribute to *O*-GlcNAcylation and to eventually develop dependence on the UPS and resistance to proteasome inhibitors by enhancing NRF1-mediated transcriptional activation of proteasome subunit genes.

A previous study reported that NRF1 is *O*-GlcNAcylated and destabilized by this modification (46), which contrasts with our observation. One possible explanation of this discrepancy would be a different antibody used in each study. The antibody used in their study for detecting *O*-GlcNAcylated protein (CTD-110.6) has been reported to cross-react with *N*-linked GlcNAcylation (47), while the one used in our study (RL-2) has higher specificity. NRF1, in its inactive state, is *N*-glycosylated and anchored in the ER membrane, resulting in the ERAD-mediated degradation (16, 19). De-*N*-glycosylation generates an active form of NRF1 in the cytoplasm and nucleus, allowing transcriptional activation of its target genes. Thus, if *N*-linked GlcNAcylation had been detected by CTD-110.6 antibody instead of *O*-GlcNAcylation, the modification would have been linked to destabilization of NRF1.

In contrast, a recent paper describes that *O*-GlcNAcylation of NRF1 by OGT increases NRF1 stability (48), which is consistent with our results. This paper suggested that HCF-1 directly interacts with NRF1 and intercalates between OGT and NRF1 for *O*-GlcNAcylation of NRF1, and our study further verified the direct interaction of NRF1 and HCF-1 using recombinant proteins (Fig. 1G). The positive impact of OGT on the activity of NRF1 has been well supported by two other independent reports that exploited siRNA screenings. OGT was included in the list of factors that confer resistance to bortezomib (49) and identified as one of the factors that promote translocation of NRF1 to the nucleus (22); both of these behaviors are consistent with the notion that we propose in this study, namely, that OGT confers bortezomib resistance on cancers by increasing NRF1 stability and thereby elevating the expression of proteasome subunit genes.

Based on the observation that the ER retention sequence of NRF1 is dispensable for the accumulation of NRF1 protein in response to the facilitation of *O*-GlcNAcylation (Fig. 7F and G), we consider that *O*-GlcNAcylation of NRF1 leads to its activation through the post-ER regulation of NRF1 activity, although we cannot completely exclude the possibility that the OGT-HCF1 complex also modulates the NRF1 activation machinery coupled with ERAD. In contrast, *N*-glycosylation of NRF1 is found to occur in the ER and has a negative impact on NRF1 activity. De-*N*-glycosylation of NRF1 constitutes the activation process of NRF1 at the ER membrane (15–17, 19–21), raising the possibility that lowering glucose availability is beneficial for NRF1 activation by limiting the amount of substrate for *N*-glycosylation. Indeed, de-*N*-glycosylation of NRF1 under no-glucose conditions has been observed to increase NRF1 activity (21). Considering our result that *O*-GlcNAcylation of NRF1 under high-glucose conditions also increases NRF1 activity, an integrated understanding of *N*-glycosylation and *O*-GlcNAcylation of NRF1 in relation to glucose availability must wait for further investigation.

Our study strongly suggests that OGT inhibition provides a promising clue to anticancer therapy by suppressing the NRF1-mediated proteasome bounce-back response. In addition, wide-ranging roles of OGT in cancer development and progression have been described (50, 51), providing a rationale for targeting OGT in anticancer therapies. Currently, no clinical data are available regarding correlations between OGT activity and therapeutic efficacy of proteasome inhibitors. It is worthwhile to examine *O*-GlcNAcylated protein levels in multiple myeloma and other cancer types in relation to their sensitivities to proteasome inhibitors.

In terms of the suppression of the proteasome bounce-back response, an alternative target of OGT has been implicated. Sharing the same DNA recognition sequence, the antioxidant response element (ARE) with NRF1, NRF2 was reported to be another regulator of proteasome subunit genes (52). A recent study has reported that a gain-of-function mutant of p53 serves as a coactivator of NRF2 and makes NRF2 coordinately activate the proteasome subunit genes through the ARE in breast cancer cells (53). Because *O*-GlcNAcylation has been shown to stabilize p53 by interfering with its interaction with the Mdm2 ubiquitin E3-ligase (54), inhibition of OGT would disrupt the p53-NRF2 pathway by destabilizing p53 and reducing the expression of proteasome subunit genes. Thus, OGT inhibition is likely to be effective in repressing the proteasome bounce-back response by suppressing NRF2 activity as well as that of NRF1.

Cellular *O*-GlcNAcylation is regulated by balancing the functions of OGT and OGA. Dysregulation of *O*-GlcNAc cycling is often found under various pathological conditions other than cancers, such as diabetes and neurodegenerative diseases (55, 56). Thiamet-G, a newly developed OGA inhibitor, has been shown to slow progression of Alzheimer's disease by an increase in tau *O*-GlcNAcylation and the reciprocal decrease in tau phosphorylation and aggregation (57). Nevertheless, it is also plausible that *O*-GlcNAcylation of NRF1 improves proteostatic failure by enhancing proteasome activity and contributing to the suppression of neurodegeneration. Our study has revealed that NRF1 transduces *O*-GlcNAcylation status into proteasome activity and suggested that *O*-GlcNAc cycling is a promising therapeutic target for diseases that feature proteostatic disturbances via the regulation of NRF1 activity.

## MATERIALS AND METHODS

### Plasmids.

pQC-mNRF1-3xFLAG was generated by inserting an mNRF1-3×FLAG cDNA fragment that was excised from pCMV-mNRF1-3xFLAG into the pQCXIP vector (Clontech). pQC-mNRF1-S-AA-3xFLAG, pQC-mNRF1-A-SS-3xFLAG, and pQC-mNRF1-A-AA-3xFLAG were generated by site-directed mutagenesis, using pQC-mNRF1-3xFLAG as a template. Construction of 3×FLAG-mNRF1 WT, 3×FLAG-mNRF1 ΔbZip, 3×FLAG-mNRF1 M1, 3×FLAG-mNRF1 M2, 3×FLAG-mNRF1 P1, mNRF1-Neh5L/AD2-His_6_ (Fr. 1; 243–430), and mNRF1-Neh6L-His_6_ (Fr. 2; 431–580) expression vectors was described previously (26, 58). pGEX4T-1-hOGT, pGEX4T-1-hHCF-1-C, pGEX4T-1-mNRF1Δ30, and pGEX4T-1-mNRF2 were generated by inserting cDNAs that encode hOGT, the C-terminal half of hHCF-1 (1012–2036), the N-terminal deletion mutant of mNRF1 (31–741), and mNRF2 into pGEX4T-1 vectors, respectively. The FLAG- and Myc-OGA expression vectors were generated by inserting hOGA/MGEA5 cDNA into a pcDNA3-FLAG and pcDNA3-Myc vector, respectively.

### Cell culture.

293F, 293T, Hep3B, HeLa, MDA-MB-231, and NCIH460 cells were maintained in high- or low-glucose Dulbecco's modified Eagle's medium (DMEM; Wako) that was supplemented with 10% fetal bovine serum (Sigma) and penicillin-streptomycin (Gibco) under 5.0% CO_2_ at 37°C. The cells that were used for experiments were cultured in low-glucose DMEM unless otherwise indicated.

### Generation of stable transformant cell lines.

Briefly, 293F and 293T cells were transduced with retrovirus expressing mouse NRF1 (mNRF1) or its mutant molecule (WT, S-AA, A-SS, and A-AA) with the 3×FLAG tag at the C terminus in a suspension with 12 μg/ml Polybrene. H460 cells were transduced with retrovirus expressing doxycycline-inducible control or *OGT* shRNA (V3SH11252-227734651; Dharmacon) in a suspension with 12 μg/ml Polybrene. One day after infection, the infected cells were replated and incubated in a selection medium that contained 2 μg/ml puromycin (Sigma). To establish stable cell lines that expressed 3×FLAG-tagged mNRF1-FL, mNRF1-ΔbZip, mNRF1-M1, and mNRF1-M2, 293F cells were transfected with pCMV-3xFLAG-mNRF1-FL, pCMV-3xFLAG-mNRF1-ΔbZip, pCMV-3xFLAG-mNRF1-M1, and pCMV-3xFLAG-mNRF1-M2, respectively. After transfection, the cells were replated and incubated in a selection medium that contained 1.5 mg/ml Geneticin (Nacalai Tesque).

### Retroviral infection.

pQC-mNRF1 (WT, S-AA, A-SS, and A-AA)-3xFLAG and pQCXIP (an empty control vector) were transfected into Plat-A cells. At 24 h after the transfection, the medium was replaced with fresh medium and cultured for another 24 h. The medium was harvested and used as the medium that contained retrovirus particles.

### Immunoblot analysis.

Immunoblot analyses were performed as described previously (59). The antibodies that were used were anti-FLAG (F7425; Sigma), anti-OGT (sc-32921; Santa Cruz), anti-HCF-1 (A301-400A; Bethyl Lab), anti-NRF1 (D5B10; Cell Signaling), anti-*O*-GlcNAc/RL2 (ab2739; Abcam), anti-6×His (9C11; Wako), anti-GST (5A7; Wako), anti-β-TrCP (D13F10; Cell Signaling), anti-NRF2 (sc-13032; Santa Cruz), and antitubulin (T9026; Sigma).

### Immunoprecipitation of NRF1 and its mutant molecules.

Soluble nuclear extracts were prepared from 293F cells that expressed mNRF1-WT, mNRF1-ΔbZip, mNRF1-M1, mNRF1-M2, and mNRF1-P1 with 3×FLAG tag at their N termini and from 293T cells that expressed mNRF1-WT, mNRF1-A-SS, mNRF1-S-AA, and mNRF1-A-AA with 3×FLAG tag at their C termini. The nuclear extracts were subjected to immunoprecipitation with anti-FLAG antibody. The immunoprecipitated samples were analyzed using immunoblot analysis.

### Recombinant protein preparation.

Fusion proteins of GST with hOGT, the C-terminal fragment of hHCF-1, mNRF1 Δ30, and mNRF2 were expressed in Escherichia coli strain BL21(DE3) and purified from soluble lysates that were prepared in PBS-T (0.1% Tween 20) by sonication. His_6_-tagged NRF1 mutants NRF1-Neh5L/AD2-His_6_ (Fr. 1; 243–430) and NRF1-Neh6L-His_6_ (Fr. 2; 431–580) were expressed in E. coli strain BL21(DE3) and purified from soluble lysates that were prepared in lysis buffer (20 mM Tris-HCl, pH 8.0, 250 mM NaCl, 10% glycerol, 40 mM imidazole, and 10 mM β-mercaptoethanol) by sonication. The soluble crude lysates were incubated with preequilibrated nickel-nitrilotriacetic acid (Ni-NTA)–agarose beads (30230; Qiagen GmbH), and the His_6_-tagged NRF1 mutants were eluted in elution buffer (20 mM Tris-HCl, pH 8.0, 250 mM NaCl, 10% glycerol, 500 mM imidazole, and 10 mM β-mercaptoethanol).

### GST pulldown assay.

GST pulldown assays were performed as described previously (60). To detect the interaction of OGT and HCF-1 with NRF1 fragments (Fr. 1 and Fr. 2), glutathione-Sepharose-immobilized GST-OGT and GST–HCF-1-C were incubated with recombinant NRF1 fragments Fr. 1 and Fr. 2 and washed extensively with PBS-T. Subsequently, pulldown proteins were eluted in Laemmli sample buffer at 94°C. Eluates were resolved by SDS-PAGE and analyzed by immunoblot assay.

### Identification of NRF1-interacting proteins.

Nuclear extract from mNRF1-3×FLAG-expressing 293F cells was prepared. The nuclear extract was subjected to anti-FLAG affinity purification. The mNRF1-3×FLAG complex was eluted using a 3×FLAG peptide according to the manufacturer's protocol (Sigma). The eluate was subjected to gel-based high-performance LC-MS/MS analysis, and NRF1-associated proteins were identified by searching the protein sequence database.

### Gel-based LC-MS/MS analysis and protein sequence database searches.

The detailed protocol for gel-based LC-MS/MS analysis and protein sequence database searches was published previously (61, 62). Briefly, after SDS-PAGE using a 5 to 20% polyacrylamide gradient gel (Oriental Instruments) and Coomassie brilliant blue staining (63), each lane in the gel was divided into 18 sections. The resulting gel blocks were treated with dithiothreitol (DTT) and acrylamide to reduce and alkylate the sulfhydryl groups. After overnight tryptic digestion, the resulting peptides in each gel block were extracted, and half of each sample was subjected to LC-MS/MS using an LTQ Orbitrap Velos mass spectrometer (Thermo Scientific). The data acquisition of every sample was performed for 60 min after a 50-min LC gradient was started; MS^1^ scans from *m*/*z* = 321 to 1,600 were carried out in the Orbitrap with the resolution set at 60,000 and the lockmass at *m*/*z* = 445.120025, followed by top-15 MS^2^ acquisition by collision-induced dissociation (CID) in the ion trap in normal-resolution mode. The settings for the MS^2^ scans were the following: minimal signal intensity required, 500; AGC target, 5,000; maximum ion injection time, 50 ms (64). The raw data files that were derived from samples in the same SDS-PAGE lane were converted together into a single MASCOT generic format file and were used for the database search by MASCOT (version 2.5.1; Matrix Science) against the mouse proteins in Swiss-Prot (July 2016) and a custom database that included mouse NRF1 protein and contaminant proteins. The peptide expectation value cutoff was set at 0.05. Protein N-terminal acetylation (+42.0106) and oxidation of methionine (+15.9949) were considered variable modifications, and propionamidated cysteine (+71.0371) was set as a fixed modification. The false discovery rates (FDR) were automatically adjusted to 1% by the MASCOT Percolator in every search.

### ChIP-seq analysis.

Sequencing libraries were prepared from 1.0 ng of DNA subjected to ChIP and input samples by a Mondrian SP+ system (Nugen) with an Ovation SP ultralow DR multiplex system (Nugen). The libraries were further purified and size-selected using an AMPure XP kit (Beckman Coulter) and were quantified by a quantitative MiSeq (qMiSeq) method (65). The samples were sequenced on a HiSeq 2500 (Illumina) that generated 101-base reads. Sequencing data were aligned with the hg19 reference genome with Bowtie2 (66), and peaks were called with MACS2 (67). ChIP-seq peak visualization was done with Integrative Genomic Viewer (68).

### ChIP assay.

ChIP assays were performed with control, HCF-1, or NRF1 siRNA-treated or nontreated HeLa cells using anti-NRF1 antibody (D5B10; Cell Signaling). The cells were treated with dimethyl sulfoxide (DMSO) or 1 μM MG132 for 4 h or with low/high glucose for 24 h and cross-linked with 1.5 mM ethylene glycol bis(succinimidyl succinate) (Thermo Scientific) for 20 min, followed by treatment with 1% formaldehyde for 10 min. The samples were then lysed and digested with micrococcal nuclease (New England BioLabs) to shear DNA. The nuclear lysis solution was incubated overnight with anti-NRF1 antibody, followed by incubation with an equal amount of Dynabeads anti-rabbit IgG (Life Technologies). Precipitated DNA was analyzed by real-time PCR using the primer sets described in Table 1.

**TABLE 1:** Primers used in ChIP analysis

Gene name	Sequence (5′–3′)	
	Forward	Reverse
*PSMA5*	GGATTCTGAGGACCAACACG	CAATAGGAAGCAGGCACAGG
*PSMD11*	CGGTGTGAGAGCGGTAAGAT	CCGATGGAGTGGAGGATGTC
*PSMD14*	GCTGCTGTTGCCTCTGTCTT	GCCTGCCTTCTGGGTCTTAC
*GATA1*	GCCTCAACTGTGTGTCCCAC	GAAGGTACTGGAAAAGTCAG

### Chemical compounds.

PugNAc, azaserine (AZA), and MG132 were purchased from Sigma. 6-Diazo-5-oxo-l-norleucine (DON), bortezomib, and b-AP15 were purchased from Wako Pure Chemicals, LC Laboratories, and LifeSensors, Inc., respectively.

### RNA purification and quantitative reverse transcription-PCR.

Total RNA samples were prepared from cells using Isogen (Nippon Gene) as previously described (60). First-strand cDNA was synthesized from 0.5 μg of total RNA using ReverTra Ace quantitative PCR reverse transcription master mix with genomic DNA remover (Toyobo). Real-time PCR was performed in triplicate for each sample with the StepOnePlus real-time PCR systems (Applied Biosystems) using primers listed in Table 2. Expression levels of rRNA were used as internal controls for normalization.

**TABLE 2:** Primers and probes used in mRNA expression analysis

Gene name	Sequence^*a*^ (5′–3′)	
	Forward	Reverse
*PSMC1*	CATGGCCACAAACCGAAT	ATCAGGCAGGGGGAACTC
*PSMC4*	GGAAGACCATGTTGGCAAAG	AAGATGATGGCAGGTGCATT
*PSMC6*	GGCAGATCGTGGGTGAAG	CGACGACAACCCACAACA
*PSMD3*	GCTGTGCAGGGCTTCTTC	GGTGTCGACGCAGCTTTT
*PSMD6*	GGCAAAGGCCGAGTACCT	ACCCAGGGCCACAGTTTT
*PSMD14*	CCGTGCTGGAGTTCCAAT	TGCCTCCACACTGACACC
*PSMB4*	TCTCGGCCAGATGGTGAT	CACATAACCGAGGAAGCT
*PSMB7*	CGGCTGTGTCGGTGTATG	GCCAGTTTTCCGGACCTT
*OGT*	CCGACTTTGGGAATCATCCT	GCTTCTGCCATCACCTTCAC
*NRF1*	TGGAACAGCAGTGGCAAGATCTCA	GGCACTGTACAGGATTTCACTTGC
*HCF-1*	GTACAACACGGCAACCAACC	CCATACTCCACCATCCCACC
*HPRT*	CCGGCTCCGTTATGGC	GGTCATAACCTGGTTCATCATCA
*HPRT* probe	FAM-CGCAGCCCTGGCGTCGTGATTA-TAMRA	

a FAM, 6-carboxyfluorescein; TAMRA, 6-carboxytetramethylrhodamine.

### siRNA transfection.

HeLa, MDA-MB-231, NCI-H460, and 293F cells were transfected with 50 nmol of siRNA using RNAiMAX (Invitrogen) according to the manufacturer's protocol. Cells were treated with MG132, bAP-15, or DMEM at 72 h after the transfection unless otherwise stated. Mission predesigned siRNAs targeting NRF1 (SASI_Hs01_00082559 and SASI_Hs01_00082561), OGT (SASI_Hs01_00141132 and SASI_Hs01_00141134), and HCF-1 (SASI_Hs01_00053487 and SASI_Hs01_00053490) were purchased from Sigma-Aldrich.

### Meta-analysis of breast and colorectal cancer cases.

TCGA data sets (TCGA; Provisional) of breast invasive carcinoma and colorectal adenocarcinoma were used for analyzing the correlation of protein abundance in the frame of cBioPortal (44, 45).

### Cell viability study.

The cell viability of MDA-MB-231 and NCIH460 cells after bortezomib treatment was determined by using a cell counting kit 8 (Nacalai Tesque) according to the manufacturer's protocol or the trypan blue exclusion test using a hemocytometer.

### Xenograft experiment.

A total of 1 × 10^6^ NCI-H460 cells expressing control or *OGT* shRNA in a doxycycline-inducible manner were subcutaneously transplanted with Matrigel to flanks of 4-week-old male BALB/c nu/nu mice. Twenty-four hours after the transplantation, the mice were administered 1 mg/ml doxycycline in 5% sucrose as drinking water. After 15 days of the transplantation (day 0 in Fig. 11B), 20 μg bortezomib in 100 μl phosphate-buffered saline (PBS) or 100 μl PBS as vehicle was directly injected into tumors whose volumes ranged from 100 to 300 mm^3^. The second injection of bortezomib was conducted after 3 days of the initial treatment (day 3 in Fig. 11B and C). Tumor volumes were measured 3 and 6 days after the initial treatment with bortezomib. All animals were housed under specific-pathogen-free conditions according to the regulations of the standards for human care and use of laboratory animals of Tohoku University and the guidelines for proper conduct of animal experiments of the Ministry of Education, Culture, Sports, Science, and Technology of Japan (http://www.mext.go.jp/b_menu/hakusho/nc/06060904.htm).

### Statistical analysis.

The quantitative data are presented as the means ± standard deviations (SD). To evaluate statistical significance, Student's *t* test was used for experiments depicted in Fig. 3, 4, and 7, and analysis of variance with Tukey-Kramer's test was used for Fig. 10 and 11.

### Accession number(s).

The data have been deposited at GEO-NCBI under the accession number GSE108856.

## Supplementary Material

Supplemental material

## References

[1] Murata S, Yashiroda H, Tanaka K 2009 Molecular mechanisms of proteasome assembly. Nat Rev Mol Cell Biol 10:104–115. doi:10.1038/nrm2630. 19165213

[2] Xie Y 2010 Structure, assembly and homeostatic regulation of the 26S proteasome. J Mol Cell Biol 2:308–317. doi:10.1093/jmcb/mjq030. 20930034

[3] Schmidt M, Finley D 2014 Regulation of proteasome activity in health and disease. Biochim Biophys Acta 1843:13–25. doi:10.1016/j.bbamcr.2013.08.012. 23994620 PMC3858528

[4] Frankland-Searby S, Bhaumik SR 2012 The 26S proteasome complex: an attractive target for cancer therapy. Biochim Biophys Acta 1825:64–76. 22037302 10.1016/j.bbcan.2011.10.003 PMC3242858

[5] Nijhawan D, Zack TI, Ren Y, Strickland MR, Lamothe R, Schumacher SE, Tsherniak A, Besche HC, Rosenbluh J, Shehata S, Cowley GS, Weir BA, Goldberg AL, Mesirov JP, Root DE, Bhatia SN, Beroukhim R, Hahn WC 2012 Cancer vulnerabilities unveiled by genomic loss. Cell 150:842–854. doi:10.1016/j.cell.2012.07.023. 22901813 PMC3429351

[6] Petrocca F, Altschuler G, Tan SM, Mendillo ML, Yan H, Jerry DJ, Kung AL, Hide W, Ince TA, Lieberman J 2013 A genome-wide siRNA screen identifies proteasome addiction as a vulnerability of basal-like triple-negative breast cancer cells. Cancer Cell 24:182–196. doi:10.1016/j.ccr.2013.07.008. 23948298 PMC3773329

[7] Weyburne ES, Wilkins OM, Sha Z, Williams DA, Pletnev AA, de Bruin G, Overkleeft HS, Goldberg AL, Cole MD, Kisselev AF 2017 Inhibition of the proteasome β2 site sensitizes triple-negative breast cancer cells to β5 inhibitors and suppresses Nrf1 activation. Cell Chem Biol 24:218–230. doi:10.1016/j.chembiol.2016.12.016. 28132893 PMC5341617

[8] Radhakrishnan SK, Lee CS, Young P, Beskow A, Chan JY, Deshaies RJ 2010 Transcription factor Nrf1 mediates the proteasome recovery pathway after proteasome inhibition in mammalian cells. Mol Cell 38:17–28. doi:10.1016/j.molcel.2010.02.029. 20385086 PMC2874685

[9] Steffen J, Seeger M, Koch A, Krüger E 2010 Proteasomal degradation is transcriptionally controlled by TCF11 via an ERAD-dependent feedback loop. Mol Cell 40:147–158. doi:10.1016/j.molcel.2010.09.012. 20932482

[10] Li X, Matilainen O, Jin C, Glover-Cutter KM, Holmberg CI, Blackwell TK 2011 Specific SKN-1/Nrf stress responses to perturbations in translation elongation and proteasome activity. PLoS Genet 7:e1002119. doi:10.1371/journal.pgen.1002119. 21695230 PMC3111486

[11] Motohashi H, O'Connor T, Katsuoka F, Engel JD, Yamamoto M 2002 Integration and diversity of the regulatory network composed of Maf and CNC families of transcription factors. Gene 294:1–12. doi:10.1016/S0378-1119(02)00788-6. 12234662

[12] Tsujita T, Peirce V, Baird L, Matsuyama Y, Takaku M, Walsh SV, Griffin JL, Uruno A, Yamamoto M, Hayes JD 2014 Transcription factor Nrf1 negatively regulates the cystine/glutamate transporter and lipid-metabolizing enzymes. Mol Cell Biol 34:3800–3816. doi:10.1128/MCB.00110-14. 25092871 PMC4187719

[13] Kobayashi A, Tsukide T, Miyasaka T, Morita T, Mizoroki T, Saito Y, Ihara Y, Takashima A, Noguchi N, Fukamizu A, Hirotsu Y, Ohtsuji M, Katsuoka F, Yamamoto M 2011 Central nervous system-specific deletion of transcription factor Nrf1 causes progressive motor neuronal dysfunction. Genes Cells 16:692–703. doi:10.1111/j.1365-2443.2011.01522.x. 21554501

[14] Lee CS, Lee C, Hu T, Nguyen JM, Zhang J, Martin MV, Vawter MP, Huang EJ, Chan JY 2011 Loss of nuclear factor E2-related factor 1 in the brain leads to dysregulation of proteasome gene expression and neurodegeneration. Proc Natl Acad Sci U S A 108:8408–8413. doi:10.1073/pnas.1019209108. 21536885 PMC3100960

[15] Radhakrishnan SK, den Besten W, Deshaies RJ 2014 p97-dependent retrotranslocation and proteolytic processing govern formation of active Nrf1 upon proteasome inhibition. Elife 3:e01856. doi:10.7554/eLife.01856. 24448410 PMC3896944

[16] Zhang Y, Lucocq JM, Yamamoto M, Hayes JD 2007 The NHB1 (N-terminal homology box 1) sequence in transcription factor Nrf1 is required to anchor it to the endoplasmic reticulum and also to enable its asparagine-glycosylation. Biochem J 408:161–172. doi:10.1042/BJ20070761. 17705787 PMC2267355

[17] Sha Z, Goldberg AL 2014 Proteasome-mediated processing of Nrf1 is essential for coordinate induction of all proteasome subunits and p97. Curr Biol 24:1573–1583. doi:10.1016/j.cub.2014.06.004. 24998528 PMC4108618

[18] Wang W, Chan JY 2006 Nrf1 is targeted to the endoplasmic reticulum membrane by an N-terminal transmembrane domain. Inhibition of nuclear translocation and transacting function. J Biol Chem 281:19676–19687. 16687406 10.1074/jbc.M602802200

[19] Zhang Y, Hayes JD 2010 Identification of topological determinants in the N-terminal domain of transcription factor Nrf1 that control its orientation in the endoplasmic reticulum membrane. Biochem J 430:497–510. doi:10.1042/BJ20100471. 20629635

[20] Zhang Y, Hayes JD 2013 The membrane-topogenic vectorial behaviour of Nrf1 controls its post-translational modification and transactivation activity. Sci Rep 3:2006. doi:10.1038/srep02006. 23774320 PMC3684815

[21] Zhang Y, Li S, Xiang Y, Qiu L, Zhao H, Hayes JD 2015 The selective post-translational processing of transcription factor Nrf1 yields distinct isoforms that dictate its ability to differentially regulate gene expression. Sci Rep 5:12983. doi:10.1038/srep12983. 26268886 PMC4534795

[22] Koizumi S, Irie T, Hirayama S, Sakurai Y, Yashiroda H, Naguro I, Ichijo H, Hamazaki J, Murata S 2016 The aspartyl protease DDI2 activates Nrf1 to compensate for proteasome dysfunction. Elife 5:e18357. doi:10.7554/eLife.18357. 27528193 PMC5001836

[23] Lehrbach NJ, Ruvkun G 2016 Proteasome dysfunction triggers activation of SKN-1A/Nrf1 by the aspartic protease DDI-1. Elife 5:e17721. doi:10.7554/eLife.17721. 27528192 PMC4987142

[24] Tomlin FM, Gerling-Driessen UIM, Liu YC, Flynn RA, Vangala JR, Lentz CS, Clauder-Muenster S, Jakob P, Mueller WF, Ordoñez-Rueda D, Paulsen M, Matsui N, Foley D, Rafalko A, Suzuki T, Bogyo M, Steinmetz LM, Radhakrishnan SK, Bertozzi CR 2017 Inhibition of NGLY1 inactivates the transcription factor Nrf1 and potentiates proteasome inhibitor cytotoxicity. ACS Cent Sci 3:1143–1155. doi:10.1021/acscentsci.7b00224. 29202016 PMC5704294

[25] Biswas M, Phan D, Watanabe M, Chan JY 2011 The Fbw7 tumor suppressor regulates nuclear factor E2-related factor 1 transcription factor turnover through proteasome-mediated proteolysis. J Biol Chem 286:39282–39289. doi:10.1074/jbc.M111.253807. 21953459 PMC3234752

[26] Tsuchiya Y, Morita T, Kim M, Iemura S, Natsume T, Yamamoto M, Kobayashi A 2011 Dual regulation of the transcriptional activity of Nrf1 by β-TrCP- and Hrd1-dependent degradation mechanisms. Mol Cell Biol 31:4500–4512. doi:10.1128/MCB.05663-11. 21911472 PMC3209242

[27] Hart GW, Housley MP, Slawson C 2007 Cycling of O-linked beta-N-acetylglucosamine on nucleocytoplasmic proteins. Nature 446:1017–1022. doi:10.1038/nature05815. 17460662

[28] Slawson C, Hart GW 2011 O-GlcNAc signaling: implications for cancer cell biology. Nat Rev Cancer 11:678–684. doi:10.1038/nrc3114. 21850036 PMC3291174

[29] Fardini Y, Dehennaut V, Lefebvre T, Issad T 2013 O-GlcNAcylation: a new cancer hallmark? Front Endocrinol (Lausanne) 4:99. 23964270 10.3389/fendo.2013.00099 PMC3740238

[30] Chikanishi T, Fujiki R, Hashiba W, Sekine H, Yokoyama A, Kato S 2010 Glucose-induced expression of MIP-1 genes requires O-GlcNAc transferase in monocytes. Biochem Biophys Res Commun 394:865–870. doi:10.1016/j.bbrc.2010.02.167. 20206135

[31] Wysocka J, Myers MP, Laherty CD, Eisenman RN, Herr W 2003 Human Sin3 deacetylase and trithorax-related Set1/Ash2 histone H3-K4 methyltransferase are tethered together selectively by the cell-proliferation factor HCF-1. Genes Dev 17:896–911. doi:10.1101/gad.252103. 12670868 PMC196026

[32] Michaud J, Praz V, James Faresse N, Jnbaptiste CK, Tyagi S, Schütz F, Herr W 2013 HCFC1 is a common component of active human CpG-island promoters and coincides with ZNF143, THAP11, YY1, and GABP transcription factor occupancy. Genome Res 23:907–916. doi:10.1101/gr.150078.112. 23539139 PMC3668359

[33] Bond MR, Hanover JA 2015 A little sugar goes a long way: the cell biology of O-GlcNAc. J Cell Biol 208:869–880. doi:10.1083/jcb.201501101. 25825515 PMC4384737

[34] Gu Y, Mi W, Ge Y, Liu H, Fan Q, Han C, Yang J, Han F, Lu X, Yu W 2010 GlcNAcylation plays an essential role in breast cancer metastasis. Cancer Res 70:6344–6351. doi:10.1158/0008-5472.CAN-09-1887. 20610629

[35] Mi W, Gu Y, Han C, Liu H, Fan Q, Zhang X, Cong Q, Yu W 2011 O-GlcNAcylation is a novel regulator of lung and colon cancer malignancy. Biochim Biophys Acta 1812:514–519. doi:10.1016/j.bbadis.2011.01.009. 21255644

[36] Zhu Q, Zhou L, Yang Z, Lai M, Xie H, Wu L, Xing C, Zhang F, Zheng S 2012 O-GlcNAcylation plays a role in tumor recurrence of hepatocellular carcinoma following liver transplantation. Med Oncol 29:985–993. doi:10.1007/s12032-011-9912-1. 21461968

[37] Baird L, Tsujita T, Kobayashi EH, Funayama R, Nagashima T, Nakayama K, Yamamoto M 2017 A homeostatic shift facilitates endoplasmic reticulum proteostasis through transcriptional integration of proteostatic stress response pathways. Mol Cell Biol 37:e00439-16. doi:10.1128/MCB.00439-16. 27920251 PMC5288573

[38] Zhang F, Su K, Yang X, Bowe DB, Paterson AJ, Kudlow JE 2003 O-GlcNAc modification is an endogenous inhibitor of the proteasome. Cell 115:715–725. doi:10.1016/S0092-8674(03)00974-7. 14675536

[39] D'Arcy P, Brnjic S, Olofsson MH, Fryknäs M, Lindsten K, De Cesare M, Perego P, Sadeghi B, Hassan M, Larsson R, Linder S 2011 Inhibition of proteasome deubiquitinating activity as a new cancer therapy. Nat Med 17:1636–1640. doi:10.1038/nm.2536. 22057347

[40] Kobayashi A, Kang MI, Okawa H, Ohtsuji M, Zenke Y, Chiba T, Igarashi K, Yamamoto M 2004 Oxidative stress sensor Keap1 functions as an adaptor for Cul3-based E3 ligase to regulate proteasomal degradation of Nrf2. Mol Cell Biol 24:7130–7139. doi:10.1128/MCB.24.16.7130-7139.2004. 15282312 PMC479737

[41] Jóźwiak P, Forma E, Bryś M, Krześlak A 2014 O-GlcNAcylation and metabolic reprograming in cancer. Front Endocrinol (Lausanne) 5:145. 25250015 10.3389/fendo.2014.00145 PMC4158873

[42] Ozcan S, Andrali SS, Cantrell JE 2010 Modulation of transcription factor function by O-GlcNAc modification. Biochim Biophys Acta 1799:353–364. doi:10.1016/j.bbagrm.2010.02.005. 20202486 PMC2881704

[43] Caldwell SA, Jackson SR, Shahriari KS, Lynch TP, Sethi G, Walker S, Vosseller K, Reginato MJ 2010 Nutrient sensor O-GlcNAc transferase regulates breast cancer tumorigenesis through targeting of the oncogenic transcription factor FoxM1. Oncogene 29:2831–2842. doi:10.1038/onc.2010.41. 20190804

[44] Cerami E, Gao J, Dogrusoz U, Gross BE, Sumer SO, Aksoy BA, Jacobsen A, Byrne CJ, Heuer ML, Larsson E, Antipin Y, Reva B, Goldberg AP, Sander C, Schultz N 2012 The cBio cancer genomics portal: an open platform for exploring multidimensional cancer genomics data. Cancer Discov 2:401–404. doi:10.1158/2159-8290.CD-12-0095. 22588877 PMC3956037

[45] Gao J, Aksoy BA, Dogrusoz U, Dresdner G, Gross B, Sumer SO, Sun Y, Jacobsen A, Sinha R, Larsson E, Cerami E, Sander C, Schultz N 2013 Integrative analysis of complex cancer genomics and clinical profiles using the cBioPortal. Sci Signal 6:pl1. doi:10.1126/scisignal.2004088. 23550210 PMC4160307

[46] Chen J, Liu X, Lü F, Ru Y, Ren Y, Yao L, Zhang Y 2015 Transcription factor Nrf1 is negatively regulated by its O-GlcNAcylation status. FEBS Lett 589:2347–2358. doi:10.1016/j.febslet.2015.07.030. 26231763

[47] Isono T 2011 O-GlcNAc-specific antibody CTD110.6 cross-reacts with N-GlcNAc2-modified proteins induced under glucose deprivation. PLoS One 6:e18959. doi:10.1371/journal.pone.0018959. 21526146 PMC3079744

[48] Han JW, Valdez JL, Ho DV, Lee CS, Kim HM, Wang X, Huang L, Chan JY 2017 Nuclear factor-erythroid-2 related transcription factor-1 (Nrf1) is regulated by O-GlcNAc transferase. Free Radic Biol Med 110:196–205. doi:10.1016/j.freeradbiomed.2017.06.008. 28625484

[49] Zhu YX, Tiedemann R, Shi CX, Yin H, Schmidt JE, Bruins LA, Keats JJ, Braggio E, Sereduk C, Mousses S, Stewart AK 2011 RNAi screen of the druggable genome identifies modulators of proteasome inhibitor sensitivity in myeloma including CDK5. Blood 117:3847–3857. doi:10.1182/blood-2010-08-304022. 21289309 PMC3083298

[50] Lynch TP, Reginato MJ 2011 O-GlcNAc transferase: a sweet new cancer target. Cell Cycle 10:1712–1713. doi:10.4161/cc.10.11.15561. 21519190

[51] Ferrer CM, Sodi VL, Reginato MJ 2016 O-GlcNAcylation in cancer biology: linking metabolism and signaling. J Mol Biol 428:3282–3294. doi:10.1016/j.jmb.2016.05.028. 27343361 PMC4983259

[52] Kwak MK, Wakabayashi N, Greenlaw JL, Yamamoto M, Kensler TW 2003 Antioxidants enhance mammalian proteasome expression through the Keap1-Nrf2 signaling pathway. Mol Cell Biol 23:8786–8794. doi:10.1128/MCB.23.23.8786-8794.2003. 14612418 PMC262680

[53] Walerych D, Lisek K, Sommaggio R, Piazza S, Ciani Y, Dalla E, Rajkowska K, Gaweda-Walerych K, Ingallina E, Tonelli C, Morelli MJ, Amato A, Eterno V, Zambelli A, Rosato A, Amati B, Wiśniewski JR, Del Sal G 2016 Proteasome machinery is instrumental in a common gain-of-function program of the p53 missense mutants in cancer. Nat Cell Biol 18:897–909. doi:10.1038/ncb3380. 27347849

[54] Yang WH, Kim JE, Nam HW, Ju JW, Kim HS, Kim YS, Cho JW 2006 Modification of p53 with O-linked N-acetylglucosamine regulates p53 activity and stability. Nat Cell Biol 8:1074–1083. doi:10.1038/ncb1470. 16964247

[55] Banerjee PS, Lagerlöf O, Hart GW 2016 Roles of O-GlcNAc in chronic diseases of aging. Mol Aspects Med 51:1–15. doi:10.1016/j.mam.2016.05.005. 27259471

[56] Bond MR, Hanover JA 2013 O-GlcNAc cycling: a link between metabolism and chronic disease. Annu Rev Nutr 33:205–229. doi:10.1146/annurev-nutr-071812-161240. 23642195 PMC10483992

[57] Yuzwa SA, Shan X, Macauley MS, Clark T, Skorobogatko Y, Vosseller K, Vocadlo DJ 2012 Increasing O-GlcNAc slows neurodegeneration and stabilizes tau against aggregation. Nat Chem Biol 8:393–399. doi:10.1038/nchembio.797. 22366723

[58] Tsuchiya Y, Taniguchi H, Ito Y, Morita T, Karim MR, Ohtake N, Fukagai K, Ito T, Okamuro S, Iemura S, Natsume T, Nishida E, Kobayashi A 2013 The casein kinase 2-nrf1 axis controls the clearance of ubiquitinated proteins by regulating proteasome gene expression. Mol Cell Biol 33:3461–3472. doi:10.1128/MCB.01271-12. 23816881 PMC3753846

[59] Sekine H, Mimura J, Oshima M, Okawa H, Kanno J, Igarashi K, Gonzalez FJ, Ikuta T, Kawajiri K, Fujii-Kuriyama Y 2009 Hypersensitivity of aryl hydrocarbon receptor-deficient mice to lipopolysaccharide-induced septic shock. Mol Cell Biol 29:6391–6400. doi:10.1128/MCB.00337-09. 19822660 PMC2786870

[60] Sekine H, Okazaki K, Ota N, Shima H, Katoh Y, Suzuki N, Igarashi K, Ito M, Motohashi H, Yamamoto M 2016 The mediator subunit MED16 transduces NRF2-activating signals into antioxidant gene expression. Mol Cell Biol 36:407–420. doi:10.1128/MCB.00785-15. 26572828 PMC4719425

[61] Ando R, Shima H, Tamahara T, Sato Y, Watanabe-Matsui M, Kato H, Sax N, Motohashi H, Taguchi K, Yamamoto M, Nio M, Maeda T, Ochiai K, Muto A, Igarashi K 2016 The transcription factor Bach2 is phosphorylated at multiple sites in murine B cells but a single site prevents its nuclear localization. J Biol Chem 291:1826–1840. doi:10.1074/jbc.M115.661702. 26620562 PMC4722461

[62] Tanaka H, Muto A, Shima H, Katoh Y, Sax N, Tajima S, Brydun A, Ikura T, Yoshizawa N, Masai H, Hoshikawa Y, Noda T, Nio M, Ochiai K, Igarashi K 2016 Epigenetic regulation of the Blimp-1 gene (Prdm1) in B cells involves Bach2 and histone deacetylase 3. J Biol Chem 291:6316–6330. doi:10.1074/jbc.M116.713842. 26786103 PMC4813568

[63] Lawrence AM, Besir HU 2009 Staining of proteins in gels with Coomassie G-250 without organic solvent and acetic acid. J Vis Exp 30:1350. 10.3791/1350 PMC3149912 19684570

[64] Kalli A, Smith GT, Sweredoski MJ, Hess S 2013 Evaluation and optimization of mass spectrometric settings during data-dependent acquisition mode: focus on LTQ-Orbitrap mass analyzers. J Proteome Res 12:3071–3086. doi:10.1021/pr3011588. 23642296 PMC3748959

[65] Katsuoka F, Yokozawa J, Tsuda K, Ito S, Pan X, Nagasaki M, Yasuda J, Yamamoto M 2014 An efficient quantitation method of next-generation sequencing libraries by using MiSeq sequencer. Anal Biochem 466:27–29. doi:10.1016/j.ab.2014.08.015. 25173513

[66] Langmead B, Salzberg SL 2012 Fast gapped-read alignment with Bowtie 2. Nat Methods 9:357–359. doi:10.1038/nmeth.1923. 22388286 PMC3322381

[67] Zhang Y, Liu T, Meyer CA, Eeckhoute J, Johnson DS, Bernstein BE, Nusbaum C, Myers RM, Brown M, Li W, Liu XS 2008 Model-based analysis of ChIP-Seq (MACS). Genome Biol 9:R137. doi:10.1186/gb-2008-9-9-r137. 18798982 PMC2592715

[68] Robinson JT, Thorvaldsdóttir H, Winckler W, Guttman M, Lander ES, Getz G, Mesirov JP 2011 Integrative genomics viewer. Nat Biotechnol 29:24–26. doi:10.1038/nbt.1754. 21221095 PMC3346182

