# Supplementary material for: O-GlcNAcylation Signal Mediates Proteasome Inhibitor Resistance in Cancer Cells by Stabilizing NRF1
Source: Mol Cell Biol. 2018 Aug 15;38(17):e00252-18. doi: 10.1128/MCB.00252-18 (PMC6094050; doi:10.1128/MCB.00252-18)
Supplement: Supplemental material [file supp_38_17_e00252-18__index.html]

Supplemental material 

# *O*-GlcNAcylation Signal Mediates Proteasome Inhibitor Resistance in Cancer Cells by Stabilizing NRF1

## Supplemental material

- Supplemental file 1 -

  Fig. S1 (NRF1 binds to promoter regions of proteasome subunit genes)

  PDF, 370K
- Supplemental file 2 -

  Table S1 (Positive correlations between OGT and majority of proteasome subunits from breast cancer cases)

  XLSX, 255K
- Supplemental file 3 -

  Table S2 (Positive correlations between OGT and majority of proteasome subunits from colorectal adenocarcinoma cases)

  XLSX, 184K
