# Supplementary material for: O-GlcNAcylation Signal Mediates Proteasome Inhibitor Resistance in Cancer Cells by Stabilizing NRF1
Source: Mol Cell Biol. 2018 Aug 15;38(17):e00252-18. doi: 10.1128/MCB.00252-18 (PMC6094050; doi:10.1128/MCB.00252-18)
Supplement: Supplemental material [file MCB.00252-18_zmb999101820s1.pdf]

Supplemental Figure S1

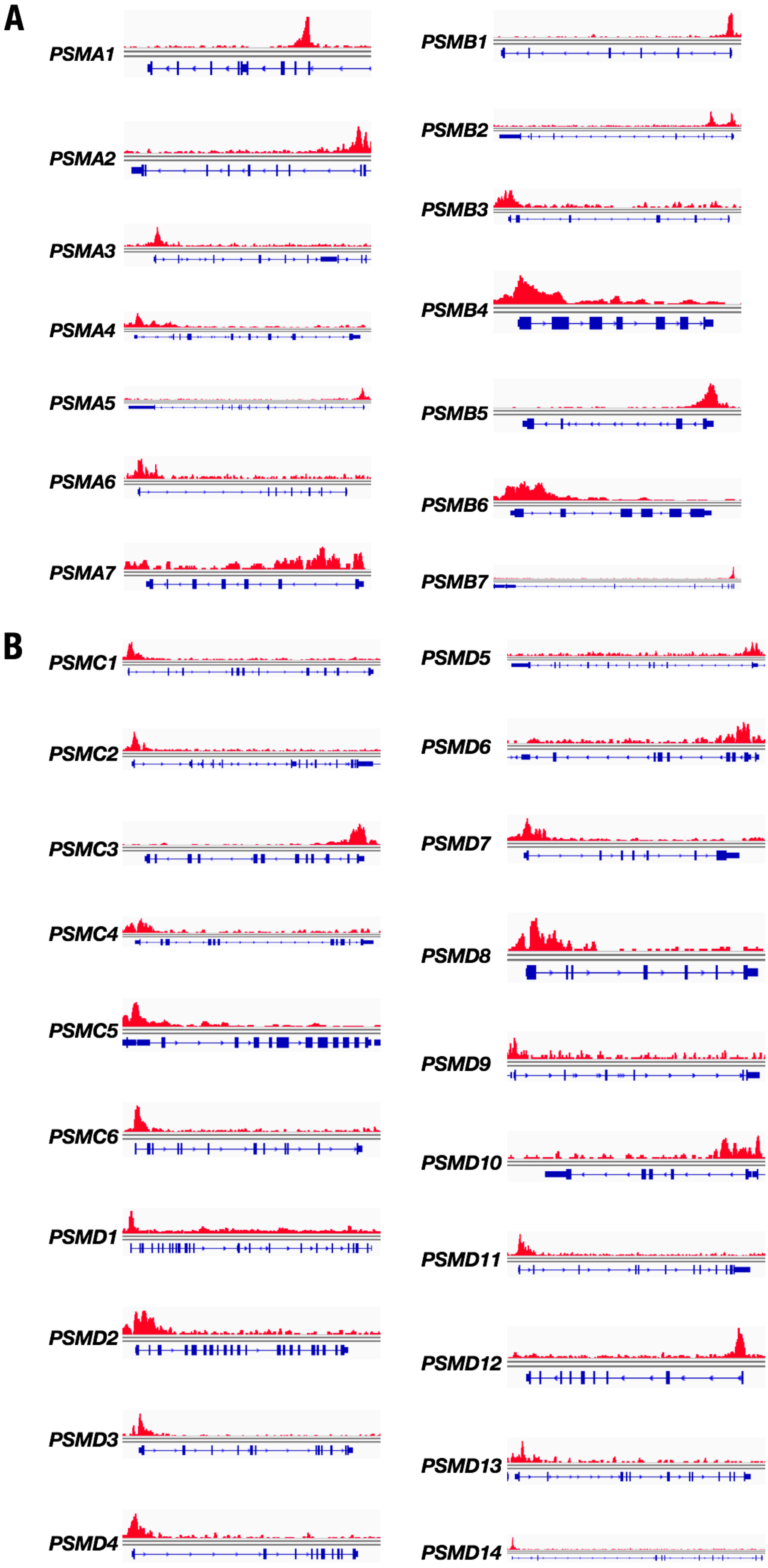

Supplemental FIG S1 NRF1 binds to promoter regions of proteasome subunit genes. (A) NRF1 ChIP-Seq peaks in PSMA and PSMB gene loci. (B) NRF1 ChIP-Seq peaks in PSMC and PSMD gene loci.
